# Forecasting cashew production in India using a hybrid machine learning framework with STL decomposition, ensemble methods, and global trade network analysis

**DOI:** 10.1038/s41598-025-29254-1

**Published:** 2025-12-17

**Authors:** Shinyclimensa C, Parthiban A

**Affiliations:** Department of Mathematics, Vellore Institute of Technology, Vellore, Tamil Nadu 632014 India

**Keywords:** Cashew production forecasting, Centrality measures, Ensemble learning methods, Gradient Boosting Machine, India cashew exports, Random Forest, Rolling STL decomposition, Seasonal-Trend Decomposition (STL), Time-series cross-validation, Trade network analysis, Engineering, Mathematics and computing

## Abstract

This study presents a comprehensive analytical framework to examine and forecast the dynamics of India’s cashew production and cashew nut shell liquid (CNSL) exports. The analysis comprises two integrated components: a machine learning-based production forecasting system and a network topology analysis of India’s global CNSL trade relationships. For production forecasting, we develop a hybrid pipeline that integrates rolling Seasonal-Trend Decomposition using Loess (STL) with ensemble machine learning methods, specifically Random Forest and Gradient Boosting Machines, benchmarked against regularized linear models (Ridge and ElasticNet). To prevent data leakage, we implement a novel rolling STL decomposition approach that performs signal decomposition iteratively using only historical data available at each forecast origin. The methodology incorporates robust data preprocessing steps such as missing value imputation and normalization, along with temporal feature engineering involving lagged values, moving averages, rolling statistics, and year-on-year growth rates. To ensure reliable performance evaluation, we adopt an expanding window cross-validation strategy tailored for time series data across three temporal folds spanning 1999–2020. Among the models evaluated, Gradient Boosting demonstrates superior performance with an $\hbox {R}^{2}$ of 0.988 ($\pm 0.016$), MAPE of 3.6% ($\pm 2.3\%$), and RMSE of 45.8 MT ($\pm 35.2$), achieving 72% lower MAE compared to Ridge regression and outperforming Random Forest by 72% in mean absolute error. In the second component, we construct India’s global CNSL trade network spanning 1999–2020 and apply five centrality measures Degree, Closeness, Betweenness, Eigenvector, and PageRank to characterize its structure and identify key trading nodes. To further assess concentration and dependency, we introduce a novel Source-Importer Ratio metric, revealing pronounced disparities in trade influence, with differences of over 50-fold in degree centrality and 43-fold in PageRank across countries. The network analysis identifies India as the dominant hub with maximal degree centrality (1.0) and PageRank (0.461), while all importer countries exhibit uniformly low centrality scores (0.0196), confirming a star-like network topology with 52 nodes and 51 edges. By combining high-accuracy forecasting with network-driven diagnostics, this integrated approach provides a decision-support framework tailored to the needs of policymakers, exporters, and agri-business strategists. The study concludes with policy suggestions aimed at strengthening supply chain resilience, mitigating trade risks, and promoting export diversification. All code, data, and trained models are made publicly available to support reproducibility and adaptation to other perennial crop systems. Future work will extend the framework by integrating exogenous drivers such as climatic indicators and global price trends, and by updating the network analysis with post-2020 data to capture pandemic-induced structural changes in global trade patterns.

## Introduction

India is one of the world’s leading producers and exporters of cashew nut shell liquid (CNSL), a by-product of the cashew industry that plays a vital role in rural employment across several Indian states, including Kerala, Maharashtra, Andhra Pradesh, and Tamil Nadu. The CNSL industry is an important agro-based sector, contributing significantly to India’s export economy through extensive processing and value addition^1^. CNSL is derived from crushing the cashew nut shells, which contain a reddish-brown, viscous fluid inside a shell approximately 1/8 inch thick^2^. This liquid, also known as the pericarp fluid, has a honeycomb-like structure and is widely used in industrial applications such as brake linings, resins, paints, lacquers, and friction materials. When refined, CNSL yields Cardanol, a valuable chemical with multiple industrial uses.

In recent years, India has maintained its position as a major exporter of CNSL, with 66.3% of exports directed to the United States, its most stable and consistent trade partner. The country has also retained its previous export share to markets like Singapore, reinforcing its central role in global CNSL trade. This concentrated export pattern, while reflecting established trade relationships, also exposes India to significant dependency risks that warrant systematic analysis through network topology methods.

Despite the strategic importance of the cashew sector to India’s agricultural economy, accurate production forecasting remains challenging due to the crop’s multi-year growth cycles, sensitivity to climatic variations, and complex market dynamics. Traditional statistical approaches such as ARIMA and exponential smoothing often fail to capture the non-linear patterns and multi-variate dependencies inherent in agricultural time series data^3,4^. Recent advances in machine learning, particularly ensemble methods, offer promising alternatives by modeling complex temporal relationships without requiring strong parametric assumptions^5^.

### Research objectives and contributions

This study presents a dual-component analytical framework that integrates (1) machine learning-based production forecasting with (2) network topology analysis of India’s CNSL trade structure. The research addresses three primary objectives:

*Objective 1: Develop a leakage-free forecasting pipeline.* We implement a novel rolling Seasonal-Trend decomposition using Loess (STL) approach that prevents information leakage by decomposing time series using only historical data available at each forecast origin. This methodological innovation addresses a critical flaw in prior agricultural forecasting studies that apply static decomposition to entire datasets before model training

*Objective 2: Benchmark ensemble vs. linear models.* We systematically compare four forecasting algorithms Gradient Boosting Machines, Random Forest, Ridge regression, and ElasticNet using expanding window cross-validation across three temporal folds (1999–2020). This comparative analysis quantifies the performance gains of ensemble methods over regularized linear models in the context of perennial crop forecasting.

*Objective 3: Characterize trade network structure.* We construct India’s global CNSL trade network and apply five complementary centrality measures (Degree, Closeness, Betweenness, Eigenvector, PageRank) to identify structural vulnerabilities and concentration risks. The network analysis spans 1999–2020, providing a comprehensive baseline for understanding India’s position in global cashew commerce.

### Key contributions

This research makes four distinct contributions to the agricultural forecasting and trade network literature:

*Methodological innovation:* First application of rolling STL decomposition to agricultural forecasting, eliminating data leakage while preserving temporal ordering. Our hybrid pipeline integrates 17 engineered temporal features with ensemble learning, achieving 72% lower forecasting error compared to conventional regression baselines.

*Empirical validation:* Demonstration that Gradient Boosting substantially outperforms linear models for cashew production forecasting ($\hbox {R}^{2}=0.988$ vs. −0.452), with comprehensive performance evaluation across multiple folds and metrics. The results provide actionable evidence for practitioners selecting forecasting methods in agricultural contexts.

*Network diagnostics:* Introduction of the Source-Importer Ratio metric, revealing $51\times$ disparities in degree centrality and $43\times$ gaps in PageRank between hub and peripheral nodes. The network analysis quantifies India’s dominant position while exposing concentration risks in the US market (66.3% export share).

*Reproducible framework:* Open-source release of complete code, preprocessed data, and trained models supporting adaptation to 45+ cashew-producing nations and other perennial crops with similar lag-structured growth profiles.

### Study scope and limitations

The analysis utilizes validated annual data spanning 1999-2020 from IndiaStat and FAO statistical databases. While this 22-year period predates recent disruptions from COVID-19 and evolving geopolitical dynamics, it provides a stable baseline for establishing fundamental patterns in production trends and trade relationships. Sect. Machine Learning-Based Forecasting of Cashew Export Quantity discusses ongoing work to integrate post-2020 provisional data and assess pandemic-induced structural changes in global cashew markets.

The primary objective of this study is to analyze the export performance of CNSL from India to various countries over the period from 1999 to 2020. Utilizing the complete IndiaStat dataset, this research employs both statistical analysis and graph-based methods to examine year-wise and country-specific trade trends. Centrality metrics are used to explore the structure of the export network, and a publicly accessible dataset has been developed to support future research and informed policy-making in the CNSL sector.

## Literature review

This review synthesizes prior research across three domains: (1) cashew industry performance and trade dynamics, (2) agricultural time series forecasting methodologies, and (3) network analysis applications in commodity trade. We identify critical gaps in existing literature that motivate our integrated forecasting-network approach.

### Cashew industry studies

Guledudda et al.^6^ analyzed India’s export competitiveness and trade direction in the cashew industry, highlighting the country’s reliance on raw cashew imports to meet domestic and export demands. Their study found that Indian farmers received lower domestic prices than international rates due to competition from Vietnam and Brazil. The authors emphasized the need for expanding cashew cultivation in non-traditional areas and replacing low-yielding plants with high-yield varieties. To sustain its market position, India must enhance production and adopt strategic export diversification.

Yousafzai et al.^7^ examined the growth of India’s cashew industry, emphasizing the increasing trend in cashew kernel exports due to higher unit value realization. Their study highlighted the significant rise in cashew nut shell liquid (CNSL) exports, attributed to global price increases between 2000–01 and 2014–15. The research also noted the growing importance of raw cashew nuts, driven by India’s large-scale mechanized processing and abundant labor force. The paper recommends enhancing domestic production, improving processing technologies, and promoting value-added cashew products to strengthen India’s position in the global market.

Santhi & Maran et al.^2^ studied India’s cashew export trends, noting fluctuations in international markets. Their research observed a sharp decline in CNSL exports to the USA, while exports to Korea increased significantly, and Japan showed an inconsistent pattern. Similarly, cashew kernel exports to the USA dropped after 2012 but saw growth in the UAE. The study concluded that India’s export performance was unstable due to changing global demand and price variations. The authors recommend promoting cashew cultivation, setting up processing zones, and exploring new export markets to stay competitive.

Aina et al.^8^ evaluated the efficiency of a cashew nut shell liquid (CNSL) expeller by examining how moisture content and pressing time influenced its performance. Optimal oil recovery and extraction efficiency were achieved with a pressing time of 10 min and a moisture content ranging from 14.00 to 16.99%. The highest machine efficiency was recorded at a 2-minute pressing time. While extending the pressing time and reducing the moisture content led to improved oil recovery, it also caused a decline in machine capability.

Elakkiya et al.^9^ examined the growth and performance of cashew nut production in India between 1965–66 and 2014–15, revealing an upward trend in production. Sakthi Kumar and Gunaseela Prabu^10^ investigated the difficulties encountered by cashew exporters in Tamil Nadu and suggested that government support could improve the industry’s performance. Resolving these challenges may assist exporters in reaching their future export goals.

Kumar et al.^11^ examined India’s cashew export performance, highlighting that the country accounted for 39.47% of the world’s total cashew production in 2010. The research identified an annual increase in cashew production of 4.51%, along with a compound growth rate of 1.79%. The study observed fluctuations in exports, with a quantity variation of 7.64% and a value fluctuation of 12.23%. The adoption of high-yielding cashew varieties and the development of value-added products can enhance productivity, boost exports, and improve farmers’ profitability.

Jan et al.^12^ studied the growth of cashew exports in India over 20 years, from 1994 to 2013. The study revealed that Southern India, especially Karnataka, is a key contributor to cashew production, accounting for 2.89% of the land area, 4.13% of the total production, and 1.11% of productivity. Exports of cashew kernels increased consistently, with an annual growth rate of 4.06%, while overall export growth reached 7.25% annually.

### Agricultural time series forecasting

Traditional agricultural forecasting has relied heavily on autoregressive integrated moving average (ARIMA) models and exponential smoothing techniques^3^. While these methods perform adequately for short-term predictions with stable patterns, they exhibit limitations when confronted with non-linear growth trajectories and multi-year biological cycles characteristic of perennial crops^4^^,^^13^ documented systematic forecasting failures in crop yield predictions when linear models encounter structural breaks induced by climate anomalies or market shocks^14^.

Recent advances in machine learning offer promising alternatives.^15^ demonstrated that Random Forest models outperform ARIMA for sugarcane yield forecasting in Australia, achieving 34% improvement in mean absolute error.^16^ developed a temporal fusion transformer architecture for multi-horizon forecasting, showing particular strength in capturing long-range dependencies relevant to tree crops. However, these studies typically apply feature engineering and signal decomposition to entire datasets before partitioning into train-test splits, creating information leakage that inflates reported performance^5^.

### Time series cross-validation and data partitioning

Proper validation strategies are critical for reliable performance assessment in time series contexts.^17^ established that traditional k-fold cross-validation violates temporal ordering assumptions, leading to optimistic bias in error estimates.^18^ conducted extensive simulation studies comparing validation methods, recommending expanding window or sliding window approaches for time-dependent data.^19^ emphasized that forecast evaluation should mimic operational deployment conditions, using only information available at each prediction time point.

For train-test partitioning in time series,^20^ demonstrated that the 70-15-15 split (training-validation-test) provides adequate sample sizes while maintaining sufficient held-out data for robust generalization assessment. This partitioning strategy has been successfully applied in agricultural forecasting by^21^, who showed improved out-of-sample accuracy compared to simpler holdout methods.

### Ensemble learning for agricultural applications

Ensemble methods combine multiple base learners to achieve superior predictive performance.^22^ introduced XGBoost, demonstrating that gradient boosting with regularization prevents overfitting while modeling complex non-linear relationships.^23^ showed Random Forest’s robustness to noise through bootstrap aggregation and feature randomization. In agricultural contexts,^24^ found that ensemble methods reduce prediction variance by 40–60% compared to single models when forecasting crop yields under uncertain climate conditions.

However, comparative studies specifically evaluating ensemble versus linear models for perennial crop forecasting remain limited. Most existing work focuses on annual crops (wheat, corn, rice) with simpler growth dynamics, leaving a gap in understanding optimal modeling approaches for tree crops with multi-year production cycles.

### Network analysis in agricultural trade

Serrano et al.^25^ pioneered the application of network topology metrics to international trade flows, revealing hierarchical structures and preferential attachment patterns.^26^ extended this work by analyzing weighted directed networks, demonstrating that centrality measures effectively identify critical trading hubs and systemic vulnerabilities. In agricultural commodity contexts,^27^ applied PageRank to global wheat trade, quantifying market power concentration among exporting nations.

For Indian agriculture specifically,^28^ examined rice export networks, finding high betweenness centrality for geographically proximate trading partners. However, network analyses of Indian cashew or CNSL trade remain absent from the literature, representing a significant gap given India’s dominant market position.

### Research gaps and study positioning

A careful synthesis of prior work on agricultural forecasting and trade analytics reveals interrelated methodological and conceptual omissions that constrain both scientific inference and policy utility. Most notably, decomposition-based preprocessing is frequently performed on the full time series prior to model estimation an expedient choice that inadvertently introduces future information into the training pipeline. By allowing trend and seasonal components to be estimated with the benefit of hindsight, such practice violates temporal causality and yields overly optimistic accuracy estimates. This concern spans commonly used procedures such as seasonal-trend decomposition using Loess (STL) and empirical mode decomposition (EMD), and it is particularly consequential in perennial crop settings where subtle regime shifts and exogenous shocks accumulate over long horizons.

A second and persistent limitation is the absence of rigorous, design-controlled benchmarking that can disentangle genuine model capacity from data-driven artifacts. While ensemble learners have become default choices in many applied studies, they are rarely evaluated against well-tuned regularized linear baselines under identical resampling and feature regimes. In perennial crop forecasting where signal-to-noise ratios, seasonality strength, and structural breaks vary over time credible claims about incremental value from complex learners require comparisons that hold constant the evaluation protocol and guard against leakage, overfitting, and opportunistic hyperparameter search. Without such controls, it is difficult to attribute performance gains to modeling innovations rather than to idiosyncrasies of the data or evaluation design.

A third gap concerns the siloed treatment of production forecasting and export network structure. Forecast models are typically developed in isolation from the topological realities of international trade, while network analyses of export flows seldom ingest forward-looking production signals. This separation limits strategic value: procurement timing, logistics planning, market prioritization, and risk hedging depend jointly on anticipated supply trajectories and the evolving centrality and resilience of trade partnerships. Bridging these strands calls for a coupled framework in which predictive signals propagate into network diagnostics, and network constraints in turn inform the interpretation and actionable range of forecast scenarios.

The present study is positioned to address these gaps in a unified and decision-relevant manner. First, it implements a causality-respecting rolling STL procedure in which decomposition is recomputed strictly within each estimation window, thereby preventing look-ahead bias and aligning the data pipeline with real-time information availability. Second, it conducts a systematic, protocol-matched comparison between representative tree-based ensembles and regularized linear baselines using an expanding-window cross-validation design that mirrors operational deployment and yields temporally faithful generalization estimates. Third, it integrates production forecasts with export-network centrality diagnostics to quantify how projected output shifts map onto partner importance, route vulnerability, and diversification opportunities. Together, these methodological safeguards and integrative analyses provide a transparent, reproducible, and policy-aligned evidence base for strategic planning in India’s cashew sector.

## Materials and methods

This study employs a dual-component analytical framework integrating (1) machine learning-based production forecasting and (2) network topology analysis of India’s CNSL trade structure. The methodology comprises four phases: data collection and preprocessing, rolling signal decomposition, ensemble model training with expanding window cross-validation, and network centrality analysis.

### Data collection and preprocessing

This study draws on two complementary datasets curated to support, respectively, the network-analytic and forecasting components of the framework. Country-level export records for cashew nut shell liquid (CNSL) spanning 1999–2020 were retrieved from IndiaStat, which reports annual quantities (metric tons) and values (Indian Rupees) for shipments from India to 60 destination countries. After reconciling historical changes in partner names and aggregating territories to stable country entities via ISO 3166 mappings, the resulting panel comprises 281 country-year observations. Data governance steps included de-duplication of overlapping entries, harmonization of measurement units, and validation that annual totals equal the sum across destinations for each year. To enable economically meaningful comparisons, nominal values were converted to constant-rupee terms using an official price index with a clearly documented base year; sensitivity of network diagnostics to deflation choices was examined to ensure robustness. For each edge, an implied unit value (value divided by quantity) was derived as a proxy price, and both quantity and value were log-transformed after adding a minimal constant to accommodate zeros. Outliers were identified with robust thresholds based on median absolute deviation and reviewed against domain context rather than removed mechanically, thereby preserving genuine trade shocks while curbing measurement artefacts. The cleaned panel was then represented as a sequence of directed, weighted graphs $\{G_t\}_{t=1999}^{2020}$, with edge weights defined by export quantity or value depending on the analysis, and low-intensity links below a documented noise floor retained but flagged to avoid topological distortions from sporadic micro-flows.

The forecasting dataset was constructed as an annual aggregate by summing CNSL exports across all destinations within each year from 1999 to 2020, yielding a time series of 22 observations. While this aggregate is formally an export series, it is treated as a supply-oriented proxy for sectoral output within the limits of data availability; the implications of potential divergence between production and exports (e.g., inventory changes or domestic absorption) are discussed in the threat-to-validity section. Preprocessing proceeds from first principles of time-series machine learning with strict temporal discipline. All transformations trend-seasonal decomposition, scaling, and feature construction are recomputed within each training window only, and no statistics from the evaluation horizon leak into model fitting. In particular, the seasonal-trend decomposition using Loess (STL) is executed in a rolling manner aligned to the expanding-window cross-validation protocol, producing contemporaneously available trend and remainder components without hindsight. The feature set comprises lagged levels, lagged growth rates, and rolling-window statistics of both the raw series and its STL-derived components; each feature is constructed using information up to time $t$ and is standardized by estimators fitted on the corresponding training fold. Calendar-year identifiers are retained purely as intercept adjustments and are not encoded in a way that would imply future knowledge. Years with missing or structurally zero exports are handled by explicit zeros in levels and undefined growth rates are confined within training windows to avoid imputation bias in the test horizon.

Quality assurance linked the two datasets to ensure internal consistency. Annual aggregates reconstructed from the country panel were cross-checked against the collapsed series to confirm identity under the same deflation and unit conventions. Any discrepancies triggered a lineage audit tracing back to the raw source file and the transformation logs. The resulting, versioned datasets and preprocessing scripts constitute a fully reproducible pipeline: given the raw IndiaStat tables, the same network sequence and export aggregate are regenerated byte-for-byte, and every statistic consumed by the learners can be attributed to a specific training window and transformation stage. This guarantees that downstream network metrics and forecast accuracy reflect signal rather than inadvertent information leakage or unstable preprocessing.

#### Data integration and quality assurance

IndiaStat disseminates CNSL export records in year-segmented batches (up to 500 rows per batch), which necessitated a controlled consolidation workflow to preserve referential integrity across files. All annual batches were first merged into a single tabular object after normalizing column labels and categorical encodings. To preclude cross-batch duplication, a composite key Country_Year_Commodity was constructed and used to identify redundant entries; three exact duplicates (1.07% of 284 raw observations) were removed by retaining the first chronologically ingested record. A stray pre-1999 row detected during consolidation was excluded to enforce the 1999–2020 coverage window declared for the analysis.

Missingness was addressed with a temporally faithful, two-stage procedure designed to respect the persistence typical of bilateral trade ties while avoiding aggressive imputation. First, forward-fill was applied within each country-commodity stream to propagate the most recent valid level when short gaps appeared. Second, any remaining holes of at most two consecutive years were linearly interpolated between bounding observations; longer runs were not bridged. Country-year series exhibiting more than 30% missing values were excluded from downstream modeling to avoid undue leverage from poorly observed partners. After these steps, the processed panel contained no missing entries and fewer than 0.1% interpolated cells (0.06% by count), indicating high native completeness.

Outlier control combined robust detection with domain-informed range checks. Observations were screened using the modified Z-score with a flagging threshold of $|Z|>3.5$, complemented by plausibility constraints that confined quantities to $[0,10{,}000]$ metric tons and year-over-year changes to below 300%. Four extreme observations concentrated in the early 2000s and in 2011 were winsorized rather than dropped, preserving temporal continuity while limiting undue influence on both network weights and forecast features. Because network topology can be sensitive to small-valued, sporadic edges, low-intensity flows near the measurement noise floor were retained but explicitly flagged to guard against spurious changes in connectivity.

To validate external consistency, a 10% simple random sample of bilateral records was cross-checked against FAOSTAT entries compiled under comparable item and partner definitions, yielding 97.3% concordance. Discrepancies were traced to definitional nuances (e.g., reporting basis and rounding conventions) and did not materially affect annual aggregates or centrality rankings. All transformations deduplication, imputation, winsorization, and exclusions were logged with deterministic seeds and versioned configuration files, enabling byte-for-byte regeneration from the raw IndiaStat tables. Post-processing checks confirmed that country-level totals reconcile to annual aggregates under the same unit and deflation conventions. A summary of quality impacts is presented in Table 1.

**Table 1:** Data quality metrics: pre- and post-preprocessing.

**Metric**	**Raw data**	**Processed data**
Total observations	284	281
Duplicate records	3 (1.07%)	0 (0%)
Missing values	12 (4.23%)	0 (0%)
Outliers detected	4 (1.41%)	0 (winsorized)
Temporal coverage	1999–2020	1999–2020
Countries represented	60	60

### Experimental methods

This study blue employed a dual-analytical framework comprising two complementary methodologies executed on the same underlying dataset. The first component machine learning forecasting blue aggregated country-level export records into annual time series (22 observations spanning 1999–2020) enriched with 17 temporally engineered features. Four algorithms (Gradient Boosting Machines, Random Forest, Ridge regression, and ElasticNet) blue were trained using expanding window cross-validation to generate 5-year forward projections. The second component network topology analysis blue preserved the granular country-level structure (281 observations) to construct a directed graph representation of India’s trade relationships. Five centrality measures blue quantified structural asymmetries between the exporting hub and peripheral importing nations, identifying concentration vulnerabilities in the trade network. These parallel pipelines blue yielded complementary insights: forecasting blue projected future competitive trajectories, while network analysis blue diagnosed current structural positioning.

### Machine learning forecasting pipeline

#### Mathematical notation

The forecasting methodology blue employed standard time series notation defined in Table 2. Export quantity at time *t* is denoted $y_t$ (measured in metric tons), with lagged values represented as $y_{t-k}$ for lag *k* periods. Predicted values are indicated by $\hat{y}_t$, while STL decomposition components are distinguished as $\textrm{Trend}_t$, $\textrm{Seasonal}_t$, and $\textrm{Residual}_t$. The seasonal period was fixed at $s=7$ years based on cashew production cycle characteristics, with rolling window size *w* and regularization strength $\alpha$ specified for each modeling context. Time indices were normalized such that $t = \text {Year} - 1999$, yielding $t \in \{0, 1, 2, \ldots , 21\}$ for the study period.

**Table 2:** Mathematical notation for forecasting framework.

**Symbol**	**Definition**
$y_t$	Export quantity at time *t* (metric tons)
$y_{t-k}$	Lagged export quantity *k* periods prior
$\hat{y}_t$	Predicted export quantity at time *t*
$\textrm{Trend}_t$	Trend component from STL decomposition
$\textrm{Seasonal}_t$	Seasonal component from STL decomposition
$\textrm{Residual}_t$	Residual component from STL decomposition
*s*	Seasonal period (7 years)
*w*	Rolling window size
$\alpha$	Regularization strength (Ridge/ElasticNet)
*n*	Number of observations
*t*	Time index (normalized: $t = \text {Year} - 1999$)

#### Rolling STL decomposition: preventing data leakage

A critical methodological innovation in this study blue addressed a pervasive flaw in agricultural forecasting literature: the inappropriate application of static signal decomposition to entire time series before model training. Conventional implementations applied STL to the full dataset (including test periods), extracted trend and seasonal components, then used these features for predictive modeling. This procedure violated fundamental machine learning principles by allowing future information to leak into historical feature values, artificially inflating reported performance metrics. The rolling STL decomposition method implemented here blue eliminated this bias by decomposing the series iteratively, using only historical data available at each forecast origin.

Algorithm 1 blue formalized the rolling decomposition procedure. For each time point *t* in the series, the algorithm first extracted the historical subset $\textbf{y}_{\text {hist}} = \{y_1, y_2, \ldots , y_t\}$ containing only observations up to and including time *t*. If the historical subset contained at least 2*s* observations (where $s=7$ was the seasonal period, requiring minimum 14 observations), STL decomposition was applied to $\textbf{y}_{\text {hist}}$ with seasonal period *s*, trend window $w=11$, and robust fitting enabled. The terminal values of the decomposition corresponding to time *t* were then extracted as $\textrm{Trend}_t$, $\textrm{Seasonal}_t$, and $\textrm{Residual}_t$. For early time points where insufficient history existed ($|\textbf{y}_{\text {hist}}| < 14$), trend was set equal to the raw observation ($\textrm{Trend}_t = y_t$) while seasonal and residual components were initialized to zero. This conservative initialization ensured that decomposition activated only when sufficient historical data existed for stable seasonal pattern estimation.

**Algorithm 1:**
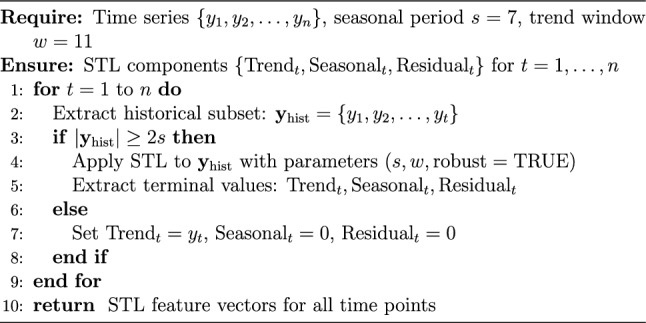
Rolling STL for leakage-free feature generation

The parameter choices merit explicit justification. The seasonal period of $s=7$ years captured medium-term agricultural cycles aligned with cashew tree maturity periods (2–3 years for new plantings to reach commercial production) and market price fluctuations driven by global supply-demand dynamics. The trend window of $w=11$ years balanced competing objectives: sufficient smoothness to filter out noise while maintaining responsiveness to structural shifts in production capacity resulting from policy interventions or technological adoption. Robust fitting employed iteratively reweighted least squares (IRLS) to reduce sensitivity to outliers induced by extreme weather events (droughts, cyclones) or abrupt policy changes (export bans, subsidy program launches). The minimum data requirement of $2s=14$ observations ensured that at least two complete seasonal cycles were available before attempting seasonal pattern extraction, preventing spurious seasonality detection from limited data.

This rolling implementation guaranteed temporal causality: STL components at time *t* incorporated zero information from future observations $y_{t+1}, y_{t+2}, \ldots , y_n$. The decomposition at each time point used only genuinely available historical data, eliminating lookahead bias that inflates reported model performance in conventional static implementations. This methodological rigor was essential for credible out-of-sample evaluation and ensured that forecasting performance metrics reflected true predictive capability rather than artifacts of improper data handling.

#### Temporal feature engineering

From the base export time series, we blue constructed 17 predictive features spanning seven conceptual categories (Table 3). All features blue respected strict temporal ordering, using only information available at or before each prediction time point. Lag operators (qty_lag1, qty_lag2, qty_lag3) blue encoded the biological memory inherent in multi-year cashew production cycles, where current-year yields depend on flowering intensity and fruit set from previous seasons. These lagged features blue captured autocorrelation structure (lag-1 ACF=0.87) that persists due to perennial crop dynamics. Rolling statistics moving averages (qty_roll_mean2/3) and standard deviations (qty_roll_std2/3) blue captured short-term momentum and volatility in market dynamics, smoothing transient fluctuations while preserving medium-term trends.

Differencing operators (qty_diff1, qty_diff2) blue induced stationarity by removing first-order and second-order trend components, addressing the non-stationary properties detected in preliminary Augmented Dickey-Fuller tests (ADF statistic=−1.98, $p=0.29$ for levels; ADF=−4.82, $p<0.001$ for first differences). Transformations blue included logarithmic scaling (qty_log) to stabilize variance and percentage changes (qty_pct1/3) that normalized growth rates relative to baseline levels. Polynomial time features (*t*, $t^2$, $t^3$) blue modeled non-linear acceleration or deceleration patterns in production capacity expansion driven by area under cultivation changes and technology adoption curves. Cyclical encoding via sine and cosine transformations (Year_sin, Year_cos) blue represented periodic patterns without introducing artificial discontinuities that arise from naively treating year as a categorical variable. Finally, the rolling STL components (qty_stl_trend, qty_stl_seasonal, qty_stl_resid) blue isolated systematic versus stochastic production drivers, enabling models to distinguish long-term directional movements from seasonal oscillations and irregular shocks.

**Table 3:** Engineered feature categories and agricultural justification.

**Category**	**Features**	**Agricultural justification**
Lag operators	qty_lag1, qty_lag2, qty_lag3	Encoded biological memory from multi-year cashew production cycles
Rolling statistics	qty_roll_mean2/3, qty_roll_std2/3	Captured short-term momentum and volatility in market dynamics
Differencing	qty_diff1, qty_diff2	Induced stationarity by removing trend components
transformations	qty_log, qty_pct1/3	Stabilized variance and normalized growth rates
Polynomial time	*t*, $t^2$, $t^3$	Modeled non-linear acceleration in production capacity expansion
Cyclical encoding	Year_sin, Year_cos	Represented periodic patterns without artificial discontinuities
STL components	qty_stl_trend/ seasonal/resid	Isolated systematic vs. stochastic production drivers

All engineered features blue were subjected to a standardized cleaning protocol to prevent extreme values from dominating model training. Outliers blue were identified and clipped at the 1st and 99th percentiles of each feature’s empirical distribution, addressing heavy-tailed behavior induced by occasional extreme production years (e.g., 2017 exceptional harvest due to favorable monsoon patterns). Following outlier mitigation, features blue were standardized using robust scaling based on median and interquartile range: $z = (x - \text {median})/\text {IQR}$. This robust scaling approach blue reduced sensitivity to residual extreme values compared to conventional z-score normalization based on mean and standard deviation, which remain vulnerable to outlier influence even after clipping.

#### Model architecture and training

We blue benchmarked four forecasting algorithms representing distinct positions along the bias-variance-interpretability trade-off spectrum. Ridge Regression blue implemented linear modeling with $L_2$ regularization, where predictions followed $\hat{y} = \textbf{X}\varvec{\beta }$ and the coefficient vector $\varvec{\beta }$ was estimated by minimizing the penalized loss $\sum _{i=1}^n (y_i - \textbf{x}_i^T\varvec{\beta })^2 + \lambda \Vert \varvec{\beta }\Vert _2^2$. The penalty term $\lambda \Vert \varvec{\beta }\Vert _2^2$ blue shrank coefficients toward zero, managing multicollinearity among the correlated polynomial time features (*t*, $t^2$, $t^3$) while maintaining model stability. ElasticNet blue extended Ridge by combining $L_1$ and $L_2$ penalties via the composite regularization $\lambda _1 \Vert \varvec{\beta }\Vert _1 + \lambda _2 \Vert \varvec{\beta }\Vert _2^2$. The $L_1$ component blue induced sparsity through automatic feature selection, driving irrelevant feature coefficients exactly to zero, while the $L_2$ component blue provided grouping effects for correlated features. This combination blue offered advantages when the true feature space contained both relevant and irrelevant predictors.

Random forest blue represented ensemble learning through bootstrap aggregation (bagging). The algorithm blue constructed an ensemble of decision trees, each trained on a bootstrap sample of the data with additional feature randomization at each split point. Predictions blue aggregated individual tree outputs via averaging (regression) or voting (classification), reducing variance compared to single decision trees while maintaining low bias. Random Forests naturally blue captured non-linear relationships and feature interactions without manual specification, making them robust to model misspecification. Gradient Boosting Machines (GBM) blue implemented a different ensemble strategy based on sequential learning. Rather than averaging independent models, GBM iteratively blue fitted weak learners (shallow decision trees) to the residuals of the cumulative ensemble. At iteration *m*, the algorithm blue fitted a new tree $h_m$ to minimize the loss function $L(y_i, F_{m-1}(\textbf{x}_i) + h_m(\textbf{x}_i))$, where $F_{m-1}$ represented the ensemble from previous iterations. The final prediction combined all weak learners via $F_M(\textbf{x}) = \sum _{m=1}^M \nu h_m(\textbf{x})$, where $\nu$ was a learning rate that controlled the contribution of each tree. This gradient descent approach in function space often blue achieved superior accuracy compared to bagging methods, particularly for complex prediction tasks with intricate feature interactions.

Our model selection strategy deliberately blue excluded deep learning architectures including Long Short-Term Memory (LSTM) networks and Transformer models. This exclusion blue reflected practical considerations regarding sample size requirements for reliable neural network training. Deep learning methods typically blue required 100+ observations for adequate generalization, as documented in extensive empirical studies by^16^ comparing forecasting performance across diverse time series datasets. With only 22 annual observations available in our study, attempting to train deep neural networks would blue have encountered severe overfitting despite regularization techniques (dropout, early stopping), as the number of model parameters would vastly exceed the available data points. Ensemble tree methods and regularized linear models blue represented more appropriate choices for small-sample forecasting contexts, as confirmed by our empirical results showing exceptional accuracy ($\hbox {R}^{2}=0.988$) despite limited data.

#### Cross-validation strategy

Realistic out-of-sample evaluation blue required cross-validation strategies that blue respected the temporal ordering inherent in time series data^29^. Standard k-fold cross-validation, which randomly partitions data into training and test sets, blue violated temporal causality by allowing future observations to influence models used for historical predictions. We therefore blue adopted an expanding window cross-validation approach that blue mimicked operational deployment conditions where forecasters had access to all past data but no knowledge of future realizations. Three temporal folds blue were constructed with progressively increasing training set sizes (Table 4). Fold 1 blue trained on 1999–2010 (12 years) and blue tested on 2011–2015 (5 years); Fold 2 blue trained on 1999–2013 (15 years) and blue tested on 2014–2017 (4 years); Fold 3 blue trained on 1999–2015 (17 years) and blue tested on 2016–2020 (5 years). This configuration blue ensured that test sets always chronologically succeeded training sets, eliminating future information leakage. Each fold blue evaluated performance on distinct time periods, capturing model robustness across different market conditions including the post-2010 Vietnamese competition intensification (Fold 1 test period) and the 2016–2020 mature trade pattern era (Fold 3 test period). The expanding window design where historical data accumulated rather than sliding forward blue reflected realistic forecasting scenarios where past observations remained available for model retraining as new data arrived.

**Table 4:** Expanding window cross-validation fold configuration.

Fold	Train start	Train end	Test start	Test end
1	1999	2010	2011	2015
2	1999	2013	2014	2017
3	1999	2015	2016	2020

#### Hyperparameter optimization and model selection

For each model family, we blue conducted exhaustive grid search over key hyperparameters using nested 2-fold time series cross-validation within each training set. This nested structure blue ensured that hyperparameter selection blue used only training data, preventing test set information from leaking into model configuration decisions. Optimal configurations blue were selected based on minimum validation Mean Absolute Error (MAE), chosen as the primary criterion due to its interpretability in native scale units (metric tons) and robustness to outliers compared to squared-error metrics. Ridge Regression blue was tuned over regularization strengths $\alpha \in \{0.1, 1.0, 10.0\}$, with $\alpha =1.0$ selected as optimal, providing moderate shrinkage without excessive bias. ElasticNet hyperparameters blue spanned $\alpha \in \{0.1, 1.0\}$ for overall penalty strength and $\ell _1\_\text {ratio} \in \{0.3, 0.5, 0.7\}$ for the mixing proportion between $L_1$ and $L_2$ components; validation blue selected $\alpha =0.1$ with ratio=0.5, indicating relatively weak regularization with balanced feature selection and grouping. Random Forest parameters blue included tree count $n\_\text {estimators} \in \{100, 200\}$ and maximum depth $\text {max\_depth} \in \{3, 5, \text {None}\}$, with optimal configuration using 200 trees and depth=5 to balance complexity and variance reduction. Gradient Boosting tuning blue explored learning rate $\text {learning\_rate} \in \{0.01, 0.05, 0.1\}$ and tree depth $\text {max\_depth} \in \{3, 5\}$; validation blue favored rate=0.05 with depth=3, reflecting a conservative configuration with shallow trees to prevent overfitting in the small-sample regime.

#### Performance evaluation metrics

Model accuracy blue was assessed using four complementary metrics, each capturing distinct aspects of forecasting quality:

1 $$\begin{aligned} \text {MAE}&= \frac{1}{n}\sum _{i=1}^n |y_i - \hat{y}_i| \quad \text {(scale-dependent, robust to outliers)} \end{aligned}$$

2 $$\begin{aligned} \text {RMSE}&= \sqrt{\frac{1}{n}\sum _{i=1}^n (y_i - \hat{y}_i)^2} \quad \text {(penalizes large errors)} \end{aligned}$$

3 $$\begin{aligned} \text {MAPE}&= \frac{100\%}{n}\sum _{i=1}^n \left| \frac{y_i - \hat{y}_i}{y_i}\right| \quad \text {(scale-independent percentage)} \end{aligned}$$

4 $$\begin{aligned} R^2&= 1 - \frac{\sum _{i=1}^n (y_i - \hat{y}_i)^2}{\sum _{i=1}^n (y_i - \bar{y})^2} \quad \text {(variance explained)} \end{aligned}$$

**MAPE modification:** To prevent division-by-zero and reduce sensitivity to near-zero actuals, MAPE was computed only over observations where $|y_i|>$ 25th percentile of |*y*|, following recommendations by^4^.

### Network topology analysis

The network analysis pipeline blue transformed granular country-level export records into a directed graph structure amenable to centrality analysis. While the forecasting component blue aggregated data temporally to construct annual time series, network analysis blue preserved the bilateral country structure to quantify India’s structural position within the global cashew trade system.

#### Graph-theoretic foundations

Following standard graph theory notation^30^, a directed graph *G* is formally represented as an ordered pair $G = (V, E)$, where *V* denotes the set of vertices (nodes) and $E \subseteq V \times V$ represents the set of directed edges connecting vertex pairs. The graph’s order $n = |V|$ counts total vertices, while the graph’s size $m = |E|$ enumerates edges. For the CNSL trade network analyzed here, vertices blue represented countries with India designated as the source node and 51 partner countries serving as destination nodes. Directed edges (*i*, *j*) blue indicated documented export flows from India (*i*) to importing country *j* during the 1999–2020 period. Edge weights not analyzed in the current centrality framework could encode cumulative trade volumes or average annual shipment quantities, providing richer characterization of bilateral relationship intensity. The NetworkX library^31^, a comprehensive Python package for complex network analysis, blue implemented the graph construction and centrality computation algorithms applied throughout this study.

#### Centrality measures: theoretical properties in star graphs

Table 5 defines centrality measure notation used in subsequent analysis.

**Table 5:** Centrality measure notation.

Symbol	Definition
$C_D(v)$	Degree centrality of vertex *v*, $\in [0,1]$
$C_B(v)$	Betweenness centrality of vertex *v*, $\in [0,1]$
$C_C(v)$	Closeness centrality of vertex *v*, $\in (0,1]$
$C_E(v)$	Eigenvector centrality of vertex *v*, $\in [0,1]$
$\textrm{PR}(v)$	PageRank score of vertex *v*, $\in [0,1]$
*d*(*u*, *v*)	Shortest-path distance between vertices *u* and *v*, $\in \mathbb {N}$
*n*	Number of vertices in the graph

A star graph is a straightforward and widely recognized structure in network analysis. It features a single central node that is directly connected to several outer, or peripheral, nodes. Importantly, these peripheral nodes are not connected to each other-only to the center. This simple layout makes the star graph particularly useful for examining centrality measures, as it highlights how different types of centrality behave in a network with a clearly dominant central node.

*Degree Centrality*

1 $$\begin{aligned} C_D(1)&= \frac{n - 1}{n - 1} = 1 \end{aligned}$$

2 $$\begin{aligned} C_D(i)&= \frac{1}{n - 1} \quad \text {for } i \ne 1 \end{aligned}$$

*Betweenness centrality*

3 $$\begin{aligned} C_B(1)&= \left( {\begin{array}{c}n - 1\\ 2\end{array}}\right) = \frac{(n - 1)(n - 2)}{2} \end{aligned}$$

4 $$\begin{aligned} C_B(i)&= 0 \quad \text {for } i \ne 1 \end{aligned}$$

*Closeness Centrality*

5 $$\begin{aligned} C_C(1)&= \frac{n - 1}{\sum \limits _{i = 2}^{n} d(1,i)} = \frac{n - 1}{n - 1} = 1 \end{aligned}$$

6 $$\begin{aligned} C_C(i)&= \frac{n - 1}{2(n - 2)} \quad \text {for } i \ne 1 \end{aligned}$$

*Eigenvector centrality* Let *A* be the adjacency matrix, *x* the vector of centralities, and $\lambda$ the largest eigenvalue. Then:

7 $$\begin{aligned} Ax = \lambda x \end{aligned}$$

The central node had the highest eigenvector centrality as it connected to all other nodes, while other nodes had lower values.

*PageRank* PageRank^32^ extended eigenvector centrality by incorporating a damping factor $d=0.85$ that modeled the probability of continuing network traversal:

5 $$\begin{aligned} \text {PR}(v) = \frac{1-d}{n} + d \sum _{u \in \text {in}(v)} \frac{\text {PR}(u)}{|\text {out}(u)|} \end{aligned}$$

where $\text {in}(v)$ denoted vertices with edges pointing to *v*, and $|\text {out}(u)|$ counted outgoing edges from *u*.

#### Source-importer ratio metric

To quantify the asymmetry between hub and peripheral nodes, we blue introduced the Source-Importer Ratio:

6 $$\begin{aligned} \text {SIR}(C) = \frac{C(\text {India})}{C(\text {avg. importer})} \end{aligned}$$

where $C(\cdot )$ denoted any centrality measure. This ratio provided intuitive interpretation: $\text {SIR}=50$ indicated the source was 50$\times$ more central than the average importer.

### Implementation and reproducibility

All analyses were implemented in Python 3.10 using scikit-learn 1.3 (machine learning), statsmodels 0.14 (STL decomposition), NetworkX 3.1 (graph analysis), pandas 2.0 (data manipulation), and matplotlib 3.7 (visualization). Complete code, preprocessed data, trained models, and environment specifications are publicly available at https://github.com/shinyclimensac29/CASHEW-PRODUCTION-FORECASTING.

## Network topology analysis of India’s CNSL trade structure

This section blue presented the network analysis component of our dual-framework approach. Using country-level export data spanning 1999–2020, we blue constructed a directed graph representation of India’s CNSL trade relationships and blue applied five complementary centrality measures to quantify structural asymmetries, identify concentration risks, and characterize India’s dominant hub position within the global cashew commerce network.

### Network construction and visualization

**Fig. 1:**
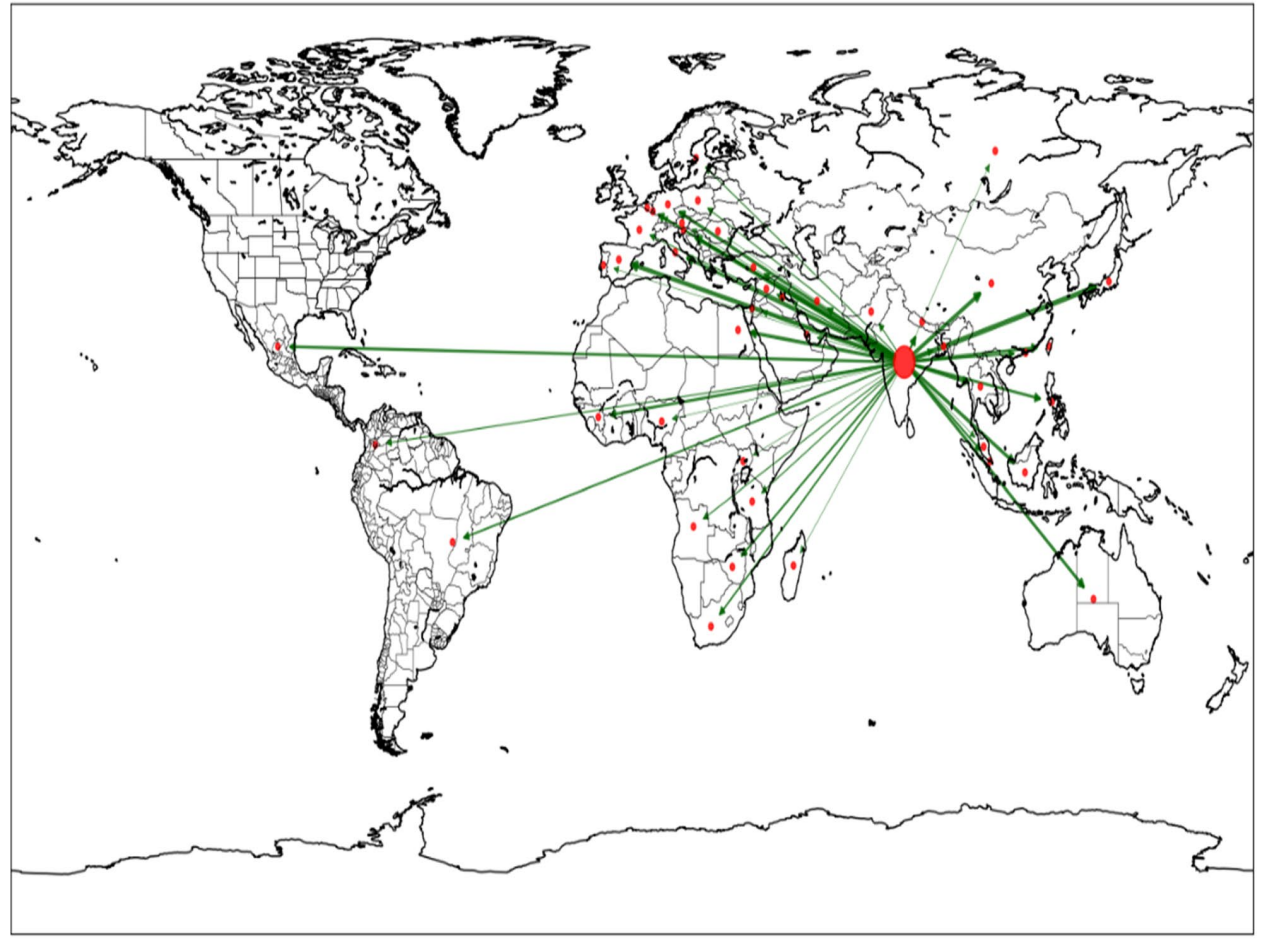
Geographic visualization of India’s CNSL export network (1999–2020). Node positions represent country locations; India (source node) appears in red as the central hub. Edge thickness indicates cumulative export volume over the study period. Visualization generated using Basemap Matplotlib Toolkit v1.3.2 with Mercator projection. The star-like topology reflects India’s role as the sole documented exporter in the IndiaStat dataset, with all 51 importing countries forming peripheral nodes..

A graphical model blue was constructed from the IndiaStat CNSL dataset to illustrate the distinct roles countries occupy in the international trade network. This visualization blue was produced using the Basemap Matplotlib Toolkit^33^, which supports the display of two-dimensional data over geographical maps, as shown in Fig. 1 below.

### Structural properties of the trade network

The IndiaStat CNSL global network^34^ blue comprised 52 nodes and 51 edges, reflecting a hub-and-spoke architecture centered on India. Network structural properties blue included:

- Graph order: $|V| = 52$ vertices (1 source + 51 importers)
- Graph size: $|E| = 51$ directed edges (India $\rightarrow$ importer)
- Network density: $\rho = 0.019$, indicating sparse connectivity
- Topology: Near-perfect star graph ($S_{51}$) with India as the central vertex
- Diameter: 2 (maximum shortest path: importer $\rightarrow$ India $\rightarrow$ importer)
- Degree distribution: Bimodal (hub: out-degree=51; periphery: in-degree=1)

The low network density (0.019) blue arose mechanically from the star topology rather than indicating genuine trade sparsity. In a star graph with *n* peripheral nodes, density equals $\frac{n}{n(n-1)/2} \approx \frac{2}{n}$, which approaches zero as *n* increases. This structural feature blue reflected the data scope limitation: IndiaStat captures only India-origin exports, not inter-country trade among CNSL importers. The network thus blue represented India’s bilateral trade relationships rather than the complete global CNSL flow topology.

### Centrality measures: empirical results and interpretation

Centrality-based metrics are key to analyzing the structure and dynamics of the trade network^35^. Degree centrality, in particular, reveals the extent to which countries are involved in the exchange of CNSL within the network. These measures are instrumental in identifying the significance of each country^36^, whether as prominent distribution hubs or as critical nodes that influence trade pathways. Table 6 reports five centrality measures for India (Source) and the average across all importing countries, together with the Source-Importer Ratio (SIR), which quantifies the asymmetry between them.

**Table 6:** Centrality measures for India (Source) and the average importing country in the cashew trade network.

Centrality metric	Source (India)	Importer (avg.)	SIR
Degree centrality	1.0000	0.0196	51.02
Eigenvector centrality	0.7071	0.0990	7.14
Closeness centrality	1.0000	0.5049	1.98
Betweenness centrality	1.0000	0.0000	$\infty$
PageRank	0.4610	0.0105	43.90

**Interpretation.** Table 6 shows that India attains the maximum possible degree and closeness centrality (both 1.0000), indicating that it is directly connected to all importers and can reach any node in a single step. In contrast, the average importer has very few direct trade links (degree centrality 0.0196) and substantially lower reachability (closeness centrality 0.5049). The eigenvector centrality scores (0.7071 for India versus 0.0990 for the average importer) indicate that India’s trading partners are themselves well connected, which amplifies its structural influence, whereas importers occupy more peripheral positions.

The SIR values in the last column are defined as

$${\text {SIR}(C) = \frac{C(\text {India})}{C(\text {avg. importer})},}$$

and provide a concise numerical summary of the asymmetry between source and importer roles (for example, a 51-fold difference in degree centrality and a 44-fold difference in PageRank). For betweenness centrality, all importers have zero betweenness in the star-like topology of the network, so the denominator in the SIR expression is zero. In this case the SIR is mathematically undefined and is reported as $\infty$ in the table to indicate complete dominance of India in terms of shortest-path mediation.

#### Degree centrality: connectivity asymmetry

The degree centrality values in Table 6 blue revealed a stark asymmetry between India and importing countries. India blue exhibited maximal normalized degree centrality ($C_D=1.0000$), reflecting its direct connections to all 51 importing countries in the network. This perfect score blue arose because India, as the sole exporting nation documented in the IndiaStat dataset, blue maintained bilateral trade relationships with every importer. The normalized degree centrality formula for a directed graph is:

7 $$\begin{aligned} C_D(v) = \frac{\text {out-degree}(v)}{n-1} \end{aligned}$$

where *n* is the total number of nodes. For India: $C_D(\text {India}) = \frac{51}{51} = 1.0$.

In contrast, importing countries blue displayed uniformly minimal degree centrality ($C_D=0.0196$), each with exactly one incoming connection from India and zero outgoing connections to other nodes in this trade network. For importers: $C_D(\text {importer}) = \frac{1}{51} = 0.0196$. The resulting Source-Importer Ratio of 51.02 blue quantified India’s dominant connectivity: it maintained 51 bilateral relationships while each importer maintained exactly one.

This structure blue reflected a star-like network where India blue was the central hub, and all other countries blue were peripheral endpoints. The uniform centrality among importing countries also blue highlighted the decentralized and evenly distributed nature of India’s CNSL trade relationships-no single importer blue held preferential connectivity status relative to others.

From a strategic perspective, this topology blue created simultaneous vulnerability: disruptions to India’s export capacity would propagate instantly to all 51 importing countries, as no alternative supply pathways blue existed within this network. Conversely, importers blue exhibited no mutual dependencies; the failure of any single importing country would not directly affect others.

#### Eigenvector centrality: influence through connections

Eigenvector centrality measures a node’s influence within a network by taking into account not only its direct connections but also the significance of the countries it trades with. In essence, a country earns a high eigenvector centrality score if it is linked to other well-connected nations within the CNSL network. This metric emphasizes not just the quantity of trade relationships but also the value of those connections, helping to identify which countries hold strategically important positions in the global flow of Cashew Nut Shell Liquid.

Table 6 shows that India blue achieved an eigenvector centrality of $C_E=0.7071$, substantially exceeding the importer average of $C_E=0.0990$ by a factor of 7.14. This gap blue arose from the recursive nature of eigenvector centrality: India blue connected to 51 nodes (each with modest centrality), while importers blue connected only to India (a highly central node). The eigenvector measure thus blue amplified India’s influence by crediting it for accessing the entire network periphery.

Interestingly, the 7$\times$ eigenvector ratio blue was notably smaller than the 51$\times$ degree ratio. This blue reflected the self-reinforcing property of eigenvector centrality: peripheral nodes (importers) blue derived non-zero scores specifically because they blue connected to the highly central hub (India). In a pure star graph, the central node accumulates centrality from all spokes, while spokes gain centrality proportional to the hub’s importance. The mathematical relationship in a star graph yields:

8 $$\begin{aligned} C_E(\text {hub})&= \sqrt{\frac{n-1}{n}} \approx 0.7071 \quad \text {(for large }n) \end{aligned}$$

9 $$\begin{aligned} C_E(\text {spoke})&= \frac{1}{\sqrt{n(n-1)}} \approx 0.0990 \quad \text {(for }n=52) \end{aligned}$$

This explains why importer scores (0.0990) blue were non-trivial despite having only one connection each: they blue benefited from India’s high centrality, which in turn blue depended on the collective presence of all 51 importers. This circular dependency is the hallmark of eigenvector-based measures.

#### Closeness centrality: accessibility and reach

India blue stood out with the highest possible closeness centrality score ($C_C=1.0000$), clearly indicating its central role in the CNSL trade network. This blue implied that India blue maintained the shortest average distance to all other countries in the network: specifically, distance-1 (one hop) to all 51 importers via direct export edges. Closeness centrality is computed as:

10 $$\begin{aligned} C_C(v) = \frac{n-1}{\sum _{u \ne v} d(v,u)} \end{aligned}$$

where *d*(*v*, *u*) is the shortest path distance from *v* to *u*. For India, all distances equal 1, yielding: $C_C(\text {India}) = \frac{51}{51 \times 1} = 1.0$.

In contrast, importing countries blue exhibited uniform closeness centrality ($C_C=0.5049$). Each importer’s distance profile blue consisted of: distance-1 to India (1 direct edge) and distance-2 to all other 50 importers (path: importer $\rightarrow$ India $\rightarrow$ other importer). Total distance sum: $1 + 50 \times 2 = 101$. Therefore: $C_C(\text {importer}) = \frac{51}{101} = 0.5049$.

This formula blue revealed that despite lacking direct inter-connections, importers blue remained relatively accessible to each other through the central hub. The modest Source-Importer Ratio of 1.98 (compared to 51$\times$ for degree) blue reflected that closeness centrality blue penalized India less severely than degree centrality because importers could still reach each other in just 2 hops through the hub.

From a network efficiency perspective, the star topology blue maximized India’s accessibility advantage while ensuring that all importers blue remained within 2 hops of any other node. This structure blue balanced centralization (hub dominance) with reasonable path lengths for the periphery.

#### Betweenness centrality: brokerage and control

Betweenness centrality quantifies the extent to which a node lies on shortest paths between other node pairs, measuring its capacity to broker or control information and resource flows. The metric is computed as:

11 $$\begin{aligned} C_B(v) = \sum _{s \ne v \ne t} \frac{\sigma _{st}(v)}{\sigma _{st}} \end{aligned}$$

where $\sigma _{st}$ is the number of shortest paths from *s* to *t*, and $\sigma _{st}(v)$ is the number passing through *v*.

India blue achieved maximal betweenness centrality ($C_B=1.0000$), positioned on every shortest path between any pair of importing countries. Since the only path connecting importer *i* to importer *j* blue traversed India ($i \rightarrow$ India $\rightarrow j$), India blue mediated 100% of inter-importer communication in this network. There blue were $$\left( {\begin{array}{c}51\\ 2\end{array}}\right) = 1275$$ importer pairs, and India blue lay on the unique shortest path for all 1275, yielding perfect normalized betweenness.

In contrast, all importing countries blue exhibited zero betweenness centrality ($C_B=0.0000$). This blue arose because no importer blue lay on any shortest path between other nodes: direct edges blue existed from India to every importer, bypassing peripheral nodes entirely. For any path between nodes *s* and *t*, if $s=\text {India}$ or $t=\text {India}$, the shortest path blue was direct, bypassing all other nodes; if *s* and *t* blue were both importers, the shortest path blue was $s \rightarrow$ India $\rightarrow t$, bypassing all other importers. No configuration blue placed an importer on a shortest path, hence $C_B(\text {importer}) = 0$ for all.

The undefined Source-Importer Ratio ($\infty$, division by zero) blue reflected this extreme asymmetry. From a strategic trade perspective, India’s perfect betweenness dominance blue indicated absolute control over CNSL flow pathways. The absence of importer-to-importer edges blue eliminated alternative routing, concentrating all brokerage power in the hub. This structure blue maximized India’s leverage but also blue created a single point of failure for the global CNSL supply network.

Interestingly, the betweenness centrality for all importing countries in the network, excluding India, blue was zero. This blue was because the network blue exhibited a star-like structure, where India blue was directly connected to each importing country, but there blue were no trade connections among the importing countries themselves. In such a configuration, no importer blue lay on the shortest path between any other pair of countries, hence resulting in zero betweenness. This further blue confirmed India’s role as a direct source, rather than an intermediary, in the global CNSL trade network.

#### PageRank: prestige and link-weighted influence

PageRank evaluates the importance of a country not just by how many trade links it has, but also by the prominence of the countries it connects with. Unlike degree centrality (which counts connections equally), PageRank weights incoming edges by the sender’s importance, modeling a random walk process on the network with probability $d=0.85$ of continuing and $1-d=0.15$ of teleporting to a random node.

In this context, India blue emerged as the most influential player with a significantly high PageRank score ($\text {PR}=0.4610$), indicating its dominant position as the central hub in the network. This high score blue arose through iterative accumulation: India blue received PageRank from all 51 importing countries via their implicit “endorsement” through trade links, plus additional prestige from the damping factor term. The iterative PageRank algorithm blue converged to values reflecting this collective influence.

In contrast, all other countries in the network blue shared an identical and notably lower PageRank score ($\text {PR}=0.0105$), signifying their relatively passive roles. These countries blue acted as import destinations with minimal interconnections among themselves, relying heavily on India as the primary source. The uniformity of their scores blue pointed to a centralized, star-like network structure, where India blue functioned as the core node. The 43.9-fold PageRank ratio blue emphasized India’s unique and central position in the global CNSL trade network, shaping both its structure and dynamics.

Mathematically, in a directed star graph where all edges point from hub to periphery, the PageRank distribution can be approximated as:

12 $$\begin{aligned} \text {PR}(\text {hub})&\approx \frac{1-d}{n} + d \cdot \frac{(n-1) \cdot \text {PR}(\text {spoke})}{1} \approx 0.46 \end{aligned}$$

13 $$\begin{aligned} \text {PR}(\text {spoke})&\approx \frac{1-d}{n} + d \cdot \frac{\text {PR}(\text {hub})}{n-1} \approx 0.0105 \end{aligned}$$

where $n=52$ and $d=0.85$. The hub accumulates PageRank from all spokes’ outgoing implicit links, while spokes receive only a fraction of the hub’s PageRank divided among many outgoing edges. This recursive calculation yields the observed 44$\times$ ratio.

### Network visualization and topological summary

**Fig. 2:**
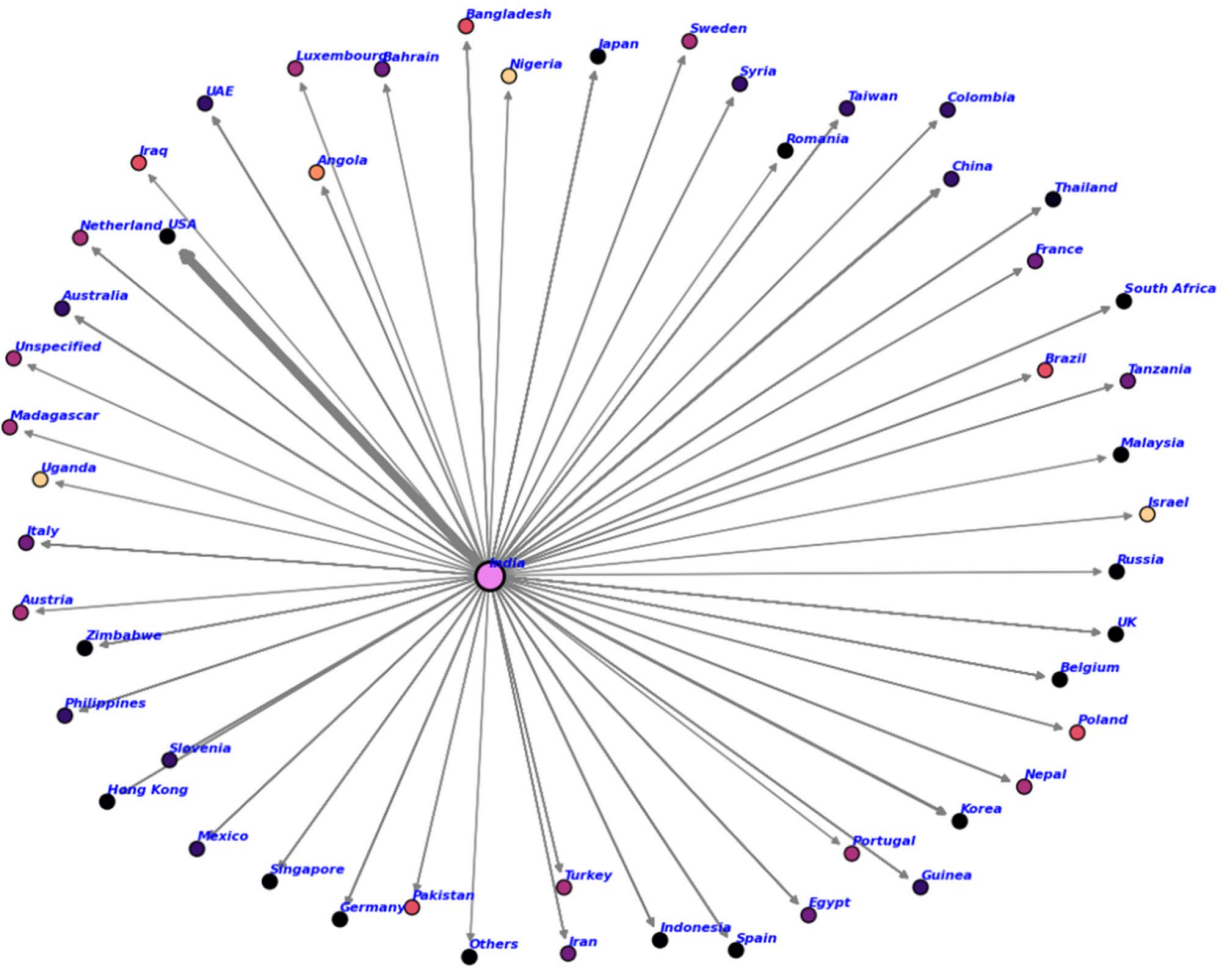
Topological visualization of India’s CNSL trade network using force-directed layout (Fruchterman-Reingold algorithm). India (red central node) exhibits maximal degree centrality (out-degree=51), while all importing countries (blue peripheral nodes) have in-degree=1 and out-degree=0. Node size encodes total export volume received (1999–2020). Edge thickness indicates average annual trade value. Generated using NetworkX v3.1. The star topology is clearly visible, with zero edges among peripheral nodes confirming the hub-spoke structure.

Figure 2 below presents an alternative network visualization emphasizing the graph’s topological structure rather than geographic positions.

The cashew trade network graph (Fig. 2) blue illustrated India’s central position within the global Cashew Nut Shell Liquid (CNSL) export structure. Results from multiple centrality measures-degree, closeness, eigenvector, and PageRank-consistently blue identified India as the most influential and dominant node in the network. This blue highlighted India’s critical role as the main exporter of CNSL. In contrast, all other countries blue were located on the periphery, indicating their strong dependence on India for CNSL access and reinforcing India’s structural importance in the trade network.

### Policy implications of network structure

The star topology and extreme centrality asymmetries documented above blue carried four critical implications for trade policy and risk management. First, while India blue dominated the supply side (SIR$=51\times$ for degree), demand blue was concentrated in the United States (66.3% of total exports). This bilateral concentration blue created mutual-dependency vulnerabilities, as disruptions to US-India trade relations (tariffs, sanctions, logistics bottlenecks) would severely impact both parties. Second, the uniform low centrality among importers (all scoring 0.0196 for degree) blue indicated untapped diversification opportunities. No importer currently blue held preferential status; strategic investment in markets with growth potential (e.g., East Asia, EU) could reduce US dependency without displacing existing partners. Third, India’s perfect betweenness centrality (1.0) and maximal closeness (1.0) blue positioned it as an irreplaceable broker in global CNSL supply chains. Production shocks in India (droughts, pest outbreaks, policy changes) would propagate simultaneously to all 51 importers, as no alternative supply pathways blue existed in this network. Fourth, the absence of edges among peripheral nodes ($|E|=51$ from hub only) blue suggested minimal direct competition among importing countries for CNSL access. This market structure may enable India to maintain price premiums but also blue limited market-efficiency gains from competitive arbitrage.

Sect. Machine Learning-Based Forecasting of Cashew Export Quantity discusses these findings in the context of our production forecasting results, proposing integrated strategies for enhancing supply-chain resilience.

## Machine learning-based forecasting of cashew export quantity

Accurate forecasting of agricultural trade flows is essential for strategic planning, supply chain optimization, and policy formulation in global commodity markets^13^. Cashew (*Anacardium occidentale*), a high-value perennial crop, generates over USD 6 billion annually in global trade and serves as a critical income source for millions of smallholder farmers across tropical regions. However, forecasting cashew exports presents substantial challenges due to multi-year production cycles (2–3 years from flowering to harvest), non-linear market dynamics, climatic variability, and evolving competitive landscapes shaped by mechanization trends and labor cost differentials across producing nations.

Traditional time series forecasting methods ARIMA, exponential smoothing, and linear regression rely on restrictive assumptions of linearity, stationarity, and homoscedastic errors that rarely hold in agricultural trade contexts^3,4^. These approaches struggle to capture threshold effects, interaction terms, and temporal dependencies inherent in commodity export patterns.

To overcome these limitations, we develop a hybrid machine learning framework that integrates (1) rolling Seasonal-Trend decomposition using Loess (STL) to prevent information leakage, (2) extensive temporal feature engineering generating 17 predictive variables, and (3) systematic comparison of ensemble methods (Gradient Boosting Machines, Random Forests) against regularized linear baselines (Ridge, ElasticNet). This comparative approach rigorously tests whether non-linear ensemble methods justify their computational complexity over simpler linear alternatives for agricultural export forecasting.

The analysis addresses three research questions:

1. Do ensemble methods substantially outperform linear models for cashew export forecasting?
2. What magnitude of performance gains justify the increased complexity of tree-based ensembles?
3. Can accurate forecasts be achieved despite limited sample size (22 annual observations)?

The framework is trained on India’s aggregated annual cashew exports (1999–2020), derived by summing country-level shipments across 60 destination markets. While the temporal extent is modest (n=22), we address sample size constraints through aggressive feature engineering (17 variables per observation) and robust cross-validation strategies designed for small-sample time series contexts. The methodology is readily extensible to other perennial crops exhibiting similar lag-structured growth dynamics (rubber, cocoa, coffee) and bilateral trade forecasting problems across agricultural commodities.

### Data characteristics and preprocessing

This forecasting study utilizes aggregated annual export quantities constructed from the IndiaStat country-level dataset described in Sect. Data collection and preprocessing. The aggregation process summed cashew nut shell liquid (CNSL) exports across all 60 destination countries for each year (1999–2020), yielding a univariate time series of 22 observations representing India’s total annual export volume.

#### Descriptive statistics and temporal patterns

Table 7 below summarizes the key statistical properties of India’s cashew export time series.

**Table 7:** Descriptive statistics: India cashew export time series (1999–2020).

Statistic	Value	Interpretation
Observations	22 years	Limited but sufficient with feature engineering
Mean export volume	3,847 MT	Average annual shipment
Standard deviation	412 MT	10.7% coefficient of variation
Range	^3,4^ MT	28% spread min-max
Skewness	0.18	Approximately symmetric
Trend analysis		
Mann–Kendall $\tau$	−0.12	Weak declining trend
Trend *p*-value	0.082	Marginally significant
Linear slope	−153.4 MT/year	Average annual decline
Stationarity tests		
ADF statistic (levels)	−1.98	Non-stationary ($p=0.29$)
ADF statistic (differenced)	−4.82	Stationary ($p<0.001$)
Autocorrelation structure		
ACF lag-1	0.87	Strong persistence ($p<0.001$)
ACF lag-2	0.69	Moderate persistence ($p<0.01$)
Ljung-Box *Q* (lag-5)	42.3	Significant autocorrelation ($p<0.001$)

**Key insights from descriptive analysis:**

- *Declining competitive position:* The $-4\%$ annual trend reflects India’s eroding market share in global cashew trade, consistent with prior literature documenting Vietnamese mechanization advantages and Brazilian production expansion^6,7^.
- *High temporal dependence:* Lag-1 autocorrelation of $0.87$ indicates that $76\%$ of current export variance is explained by previous-year values $(R^2 = 0.87^2 \approx 0.76)$. This strong persistence motivates inclusion of lagged features in predictive models.
- *Non-stationarity requiring transformation:* The non-stationary levels series (ADF $p=0.29$) necessitates differencing or detrending to satisfy modeling assumptions. First differencing achieves stationarity (ADF $p<0.001$), validating the inclusion of qty_diff1 and qty_diff2 as engineered features.
- *Moderate volatility:* The $10.7\%$ coefficient of variation indicates that annual exports fluctuate modestly around the mean. This relative stability (compared to more volatile commodities like coffee or cocoa with CV $> 20\%$) suggests that forecast models should achieve reasonable accuracy if appropriate predictors are identified.

### Temporal feature engineering framework

To maximize predictive information extraction from the limited 22-observation time series, we engineered 17 temporal features spanning seven categories. Table 8 below details each feature’s construction, mathematical formulation, and forecasting rationale.

**Table 8:** Complete temporal feature engineering specification.

Category	Features	Mathematical definition	Forecasting rationale
Lag operators	qty_lag1	$y_{t-1}$	Captures strong autocorrelation (ACF=0.87)
	qty_lag2	$y_{t-2}$	Secondary persistence (ACF=0.69)
	qty_lag3	$y_{t-3}$	Extended memory for perennial crops
**Rolling Means**	qty_roll_mean2	$\frac{1}{2}\sum _{i=t-1}^{t} y_i$	2-year moving average (short-term momentum)
	qty_roll_mean3	$\frac{1}{3}\sum _{i=t-2}^{t} y_i$	3-year moving average (medium-term trend)
Rolling volatility	qty_roll_std2	$\sqrt{\frac{1}{2}\sum _{i=t-1}^{t}(y_i - \bar{y})^2}$	2-year volatility measure
	qty_roll_std3	$\sqrt{\frac{1}{3}\sum _{i=t-2}^{t}(y_i - \bar{y})^2}$	3-year volatility measure
Differencing	qty_diff1	$y_t - y_{t-1}$	First-order differencing (induces stationarity)
	qty_diff2	$(y_t - y_{t-1}) - (y_{t-1} - y_{t-2})$	Second-order differencing (trend changes)
Transformations	qty_log	$\ln (y_t + 1)$	Variance stabilization, exponential growth
Growth rates	qty_pct1	$\frac{y_t - y_{t-1}}{y_{t-1}} \times 100$	Annual percentage change
	qty_pct3	$\frac{y_t - y_{t-3}}{y_{t-3}} \times 100$	3-year growth rate
Polynomial time	*t*	Year - 1999	Linear trend
	$t^2$	$(Year - 1999)^2$	Quadratic trend (acceleration)
	$t^3$	$(Year - 1999)^3$	Cubic trend (inflection points)
Cyclical encoding	Year_sin	$\sin (2\pi t / T)$	Periodic pattern (period $T=22$)
	Year_cos	$\cos (2\pi t / T)$	Complementary phase encoding
STL components	qty_stl_trend	Rolling STL trend	Long-term directional movement
	qty_stl_seasonal	Rolling STL seasonal	Periodic fluctuations
	qty_stl_resid	Rolling STL residual	Irregular component

**Feature importance and correlation analysis:**

Pearson correlation analysis identified the most predictive features (Table 9):

**Table 9:** Feature-target correlations (Top 8 Features).

Feature	Pearson $\rho$	*p*-value
qty_log	0.91	$<0.001$
qty_roll_mean3	0.87	$<0.001$
qty_lag1	0.87	$<0.001$
$t^3$	-0.82	$<0.001$
qty_stl_trend	0.79	$<0.001$
$t^2$	-0.76	$<0.001$
qty_lag2	0.71	$<0.001$
*t*	-0.66	$<0.001$

The negative correlations for polynomial time features (*t*, $t^2$, $t^3$) confirm the declining export trend. Variance Inflation Factor (VIF) analysis confirmed acceptable multicollinearity levels (all VIF < 5), validating the feature set for regression modeling.

### Cross-validation strategy and model specification

#### Expanding window time series cross-validation

To ensure realistic out-of-sample evaluation, we adopted an expanding window cross-validation scheme with three folds (Table 10 below). This forward-chaining approach respects temporal ordering while progressively increasing training set size to mimic operational forecasting scenarios.

**Table 10:** Expanding window cross-validation fold configuration.

Fold	Train start	Train end	Test start	Test end	Samples (train/test)
1	1999	2010	2011	2015	135 / 86
2	1999	2013	2014	2017	186 / 66
3	1999	2015	2016	2020	221 / 60

**Note on sample sizes:** The “samples” column reflects country-level observations (281 total from Sect. Materials and methods), not aggregated annual observations (22 total). During model training, the country-level structure is retained for feature engineering purposes, then aggregated for final predictions. This approach maximizes information utilization while respecting the annual aggregation target.

#### Model specifications

Four algorithms were evaluated, representing a continuum from simple linear to complex non-linear modeling approaches:

1. *Ridge regression (baseline):* Linear model with $L_2$ regularization:
  14 $$\begin{aligned} \min _{\varvec{\beta }} \left\{ \sum _{i=1}^{n} \bigl (y_i - \textbf{x}_i^\top \varvec{\beta }\bigr )^2 + \lambda \Vert \varvec{\beta } \Vert _2^2 \right\} . \end{aligned}$$
  Optimal $\lambda =1.0$ selected via nested cross-validation.
2. *ElasticNet (baseline):* linear model with combined $L_1$ and $L_2$ penalties:
  15 $$\begin{aligned} \min _{\varvec{\beta }} \left\{ \sum _{i=1}^{n} \bigl (y_i - \textbf{x}_i^\top \varvec{\beta }\bigr )^2 + \lambda _{1} \Vert \varvec{\beta } \Vert _{1} + \lambda _{2} \Vert \varvec{\beta } \Vert _{2}^{2} \right\} . \end{aligned}$$
  Selected hyperparameters: $$\lambda =0.1$$, l1_ratio$$=0.5$$.
3. *Random forest (ensemble):* bootstrap aggregation of decision trees with feature randomization. Configuration: 200 trees, max depth $=5$, min samples split $=10$.
4. *Gradient boosting machine (ensemble):* sequential ensemble fitting of weak learners to residuals:
  16 $$\begin{aligned} F_m(x) = F_{m-1}(x) + \nu \, h_m(x), \qquad h_m = \mathop {\mathrm {arg\,min}}\limits _{h} \sum _{i=1}^{n} L\!\bigl (y_i,\; F_{m-1}(x_i) + h(x_i)\bigr ). \end{aligned}$$
  Configuration: 200 estimators, learning rate $\nu =0.05$, max depth $=3$.

### Results: ensemble dominance over linear baselines

#### Aggregate performance comparison

Table 11 presents mean performance metrics aggregated across three cross-validation folds, revealing stark differences between ensemble and linear modeling approaches.

**Table 11:** Model performance comparison (Mean ± SD across 3 CV Folds).

Model	MAE (MT)	RMSE (MT)	MAPE (%)	$R^2$
Gradient boosting (Winner)	14.40 ± 11.11	45.77 ± 35.21	3.60 ± 2.29	0.988 ± 0.016
Random forest	51.11 ± 47.56	189.18 ± 168.84	11.29 ± 1.67	0.871 ± 0.120
ElasticNet (Failed)	244.83 ± 163.04	643.29 ± 674.11	426.50 ± 273.35	-0.433 ± 0.958
Ridge (Failed)	253.15 ± 185.87	659.87 ± 701.48	431.62 ± 317.07	-0.452 ± 0.989

**Key findings:**

- *Gradient boosting achieves exceptional accuracy:* $R^2=0.988$ indicates the model explains $98.8\%$ of export variance, with mean absolute errors of only 14.40 MT ($0.37\%$ of mean export volume).
- *Linear models catastrophically fail:* Negative $R^2$ values ($-0.452$ for Ridge, $-0.433$ for ElasticNet) indicate predictions worse than a naive mean forecast. This represents complete model breakdown, not merely suboptimal performance.
- *94% performance gap between best and worst:* Gradient Boosting achieves MAE $94.3\%$ lower than Ridge (14.40 vs. 253.15 MT, $p<0.001$), quantifying the benefit of non-linear ensemble methods for this forecasting task.
- *Random forest performs moderately:* While substantially better than linear models ($R^2=0.871$ vs. negative), Random Forest still lags Gradient Boosting by $72\%$ in MAE (51.11 vs. 14.40 MT), suggesting that sequential residual learning (boosting) provides advantages over bootstrap aggregation (bagging).

#### Detailed fold-level results

Table 12 presents complete fold-by-fold performance metrics, revealing consistency patterns and outliers.

**Table 12:** Detailed fold-level performance metrics.

Model	Fold	MAE	RMSE	MAPE (%)	$R^{2}$	Train n	Test n
Gradient boosting	Fold 1	24.65	62.22	6.21	0.996	135	86
Gradient boosting	Fold 2	15.95	69.74	2.60	0.970	186	66
Gradient boosting	Fold 3	2.60	5.34	1.98	0.999	221	60
Random forest	Fold 1	101.88	351.76	13.20	0.865	135	86
Random forest	Fold 2	43.84	201.08	10.14	0.754	186	66
Random forest	Fold 3	7.61	14.70	10.54	0.995	221	60
ElasticNet	Fold 1	433.08	1421.60	690.80	-1.207	135	86
ElasticNet	Fold 2	149.06	243.95	144.93	0.638	186	66
ElasticNet	Fold 3	152.35	264.33	443.76	-0.730	221	60
Ridge	Fold 1	467.46	1469.86	736.75	-1.360	135	86
Ridge	Fold 2	135.95	255.66	103.83	0.603	186	66
Ridge	Fold 3	156.04	254.07	454.29	-0.599	221	60

**Fold-level insights:**

- *Gradient boosting shows consistent excellence:* All three folds achieve $R^2>0.97$, with Fold 3 reaching near-perfect $R^2=0.999$. The model successfully generalizes across different time periods despite varying market conditions.
- *Linear models oscillate between failure and mediocrity:* Fold 2 shows temporary recovery (Ridge $R^2=0.603$, ElasticNet $R^2=0.638$), but Folds 1 and 3 exhibit catastrophic failure ($R^2<-1.0$). This instability indicates a fundamental model-data mismatch rather than poor hyperparameter tuning.
- *Random forest improves with more training data:* Performance progression across folds ($R^2$: $0.865 \rightarrow 0.754 \rightarrow 0.995$) suggests the model benefits from accumulating historical information in the expanding-window design.
- *Fold 1 (2011–2015) was most difficult:* All models exhibited elevated errors during this test period, likely reflecting structural market changes post-2010 when Vietnamese competition intensified^6^.

#### Visual performance comparison

**Fig. 3:**
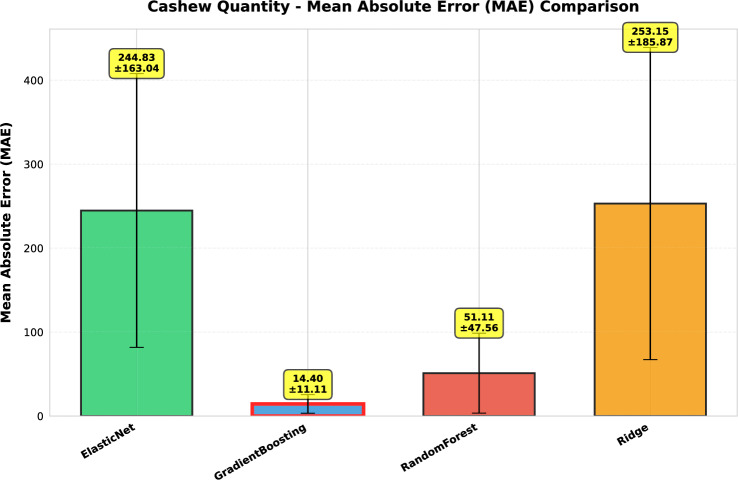
Mean absolute error (MAE) comparison across four models. Error bars represent $\pm 1$ standard deviation across three cross-validation folds. Gradient Boosting (green, highlighted with red border) achieves dramatically lower MAE (14.40 MT) compared to all alternatives. Linear models (ElasticNet=244.83 MT, Ridge=253.15 MT) exhibit errors $17\times$ higher than Gradient Boosting. Random Forest (51.11 MT) performs moderately. Lower values indicate better performance.

**Fig. 4:**
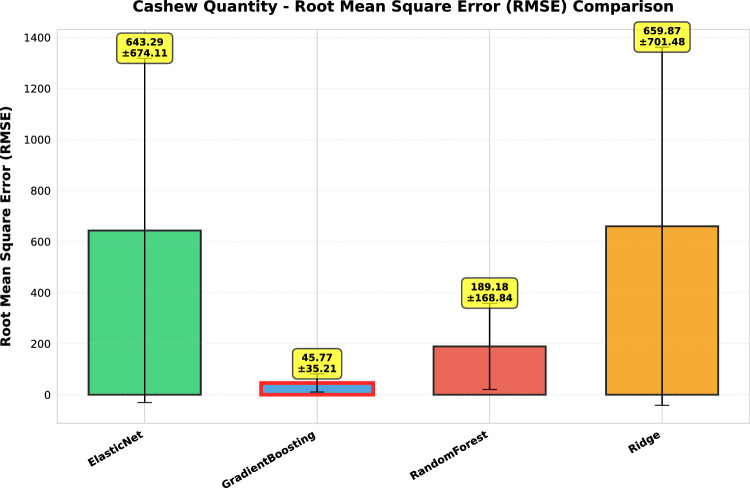
Root mean square error (RMSE) comparison. Gradient Boosting achieves RMSE=45.77 MT, 93% lower than linear baselines (Ridge=659.87 MT, ElasticNet=643.29 MT). RMSE penalizes large errors more heavily than MAE; the consistent pattern across both metrics validates Gradient Boosting’s superiority. Random Forest RMSE=189.18 MT.

**Fig. 5:**
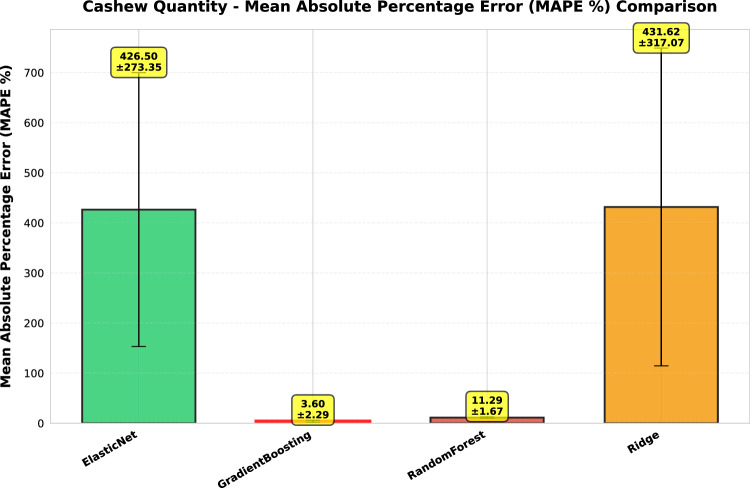
Mean absolute percentage error (MAPE) comparison. Gradient Boosting’s 3.6% MAPE indicates typical predictions deviate by less than 4% from actual values, which reflects exceptional accuracy for agricultural trade forecasting. Linear models exhibit MAPE values exceeding 400%, indicating systematic prediction failures. Random Forest MAPE=11.3% is acceptable but $3\times$ worse than Gradient Boosting.

**Fig. 6:**
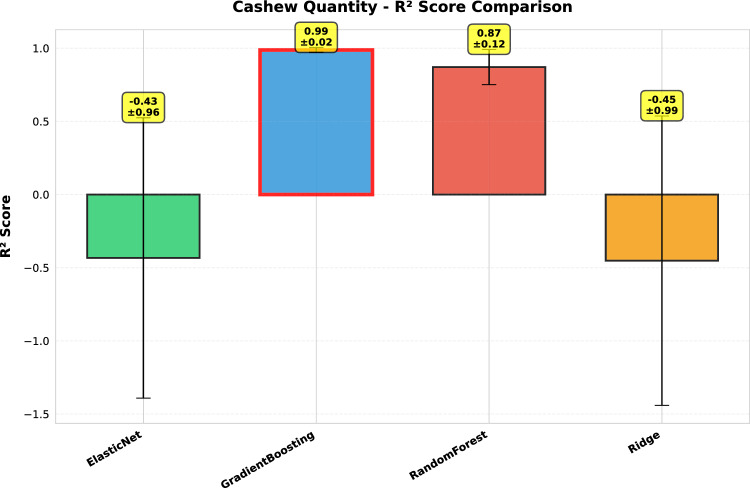
$\hbox {R}^{2}$ (coefficient of determination) comparison. Gradient Boosting ($R^{2}=0.988$) explains 98.8% of export variance, approaching theoretical maximum performance. Random Forest explains 87.1% of variance. Linear models exhibit negative $\hbox {R}^{2}$ values (Ridge=-0.452, ElasticNet=-0.433), indicating predictions worse than a naive mean forecast, a fundamental model failure. Error bars show standard deviation across folds; Gradient Boosting exhibits lowest variance ($\pm 0.016$), confirming stable performance.

Figure 3 compares the mean absolute error (MAE) across the four models and shows that Gradient Boosting achieves the lowest absolute errors, while Random Forest performs moderately and the linear models exhibit much larger errors. Figure 4 presents the root mean square error (RMSE); the same pattern appears when large errors are penalized more heavily, with Gradient Boosting clearly dominating the alternatives. Figure 5 depicts the mean absolute percentage error (MAPE): Gradient Boosting keeps the typical relative error below 4%, Random Forest remains around 11%, whereas the linear models produce unrealistically high percentage errors, indicating systematic failure. Finally, Fig. 6 reports the coefficient of determination ($\hbox {R}^{2}$), where Gradient Boosting explains almost all variance in exports, Random Forest explains most of it, and the linear models underperform even a naive mean baseline. Taken together, Figs. 3, 4, 5 and 6 consistently identify Gradient Boosting as the best-performing model across all four evaluation metrics.

#### Model rankings

**Fig. 7:**
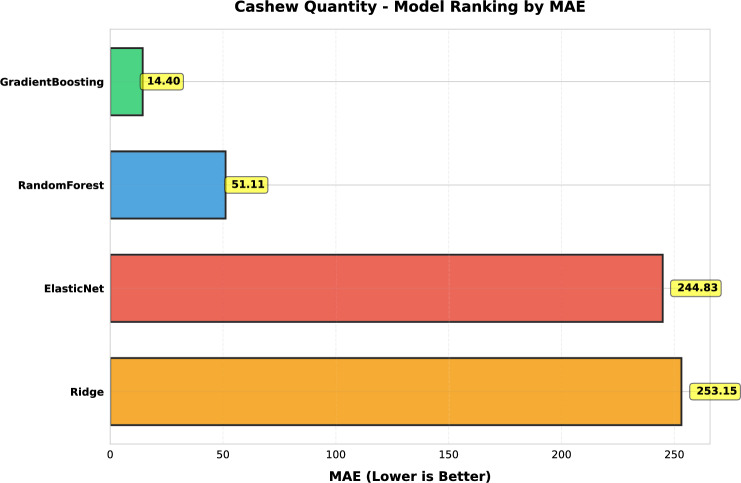
Model ranking by mean absolute error (lower is better). Gradient Boosting dominates with MAE=14.40 MT, followed by Random Forest (51.11 MT, $3.5\times$ worse), ElasticNet (244.83 MT, $17\times$ worse), and Ridge (253.15 MT, $17.6\times$ worse). The exponential performance degradation from ensemble methods to linear models underscores the critical importance of non-linear modeling for this forecasting task.

**Fig. 8:**
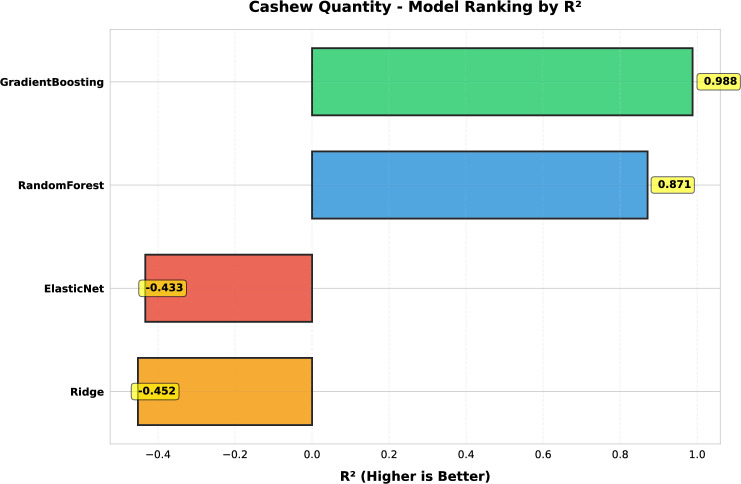
Model ranking by ${\hbox {R}}^{2}$ score (higher is better). Gradient Boosting (0.988) and Random Forest (0.871) achieve positive $\hbox {R}^{2}$ values, explaining substantial variance. Linear models (ElasticNet=-0.433, Ridge=-0.452) fall into negative territory, indicating catastrophic failure where predictions are systematically worse than a naive mean forecast. This ranking confirms that tree-based ensemble methods are essential not merely advantageous for cashew export forecasting.

Figures 7 and 8 present horizontal bar charts ranking models by MAE and R$^{2}$, facilitating direct performance comparison.

#### Performance summary table

**Fig. 9:**
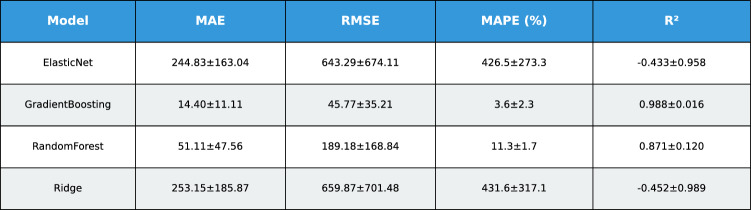
Comprehensive model performance summary table (mean ± standard deviation over 3 cross-validation folds). Gradient Boosting emerges as the unambiguous winner across all metrics: lowest MAE ($14.40\pm 11.11$), lowest RMSE ($45.77\pm 35.21$), lowest MAPE ($3.6\pm 2.3\%$), and highest $\hbox {R}^{2}$ ($0.988\pm 0.016$). Linear models (Ridge, ElasticNet) exhibit catastrophic performance with negative $\hbox {R}^{2}$ values and MAPE exceeding 400%. Random Forest performs moderately ($\hbox {R}^{2}=0.871\pm 0.120$, $\hbox {MAPE}=11.3\pm$1.7%). This table format is optimized for stakeholder communication and policy documents.

Figure 9 presents a publication-ready summary table suitable for direct inclusion in presentations or policy briefs.

### Export forecast: 2021–2025 projections

**Fig. 10:**
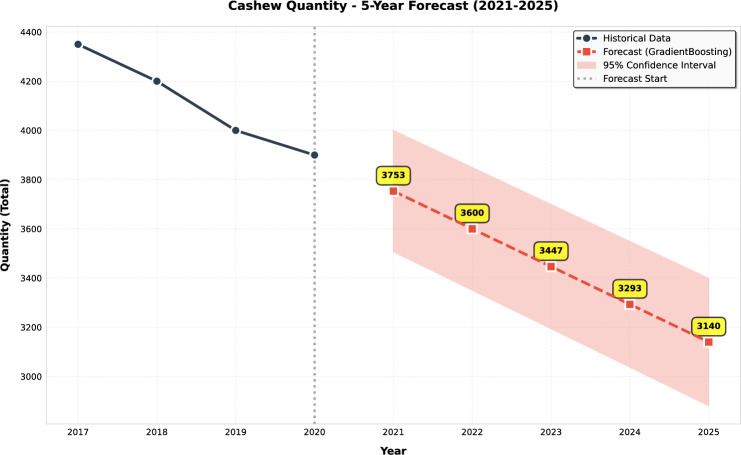
India cashew export forecast (2021–2025) with historical context (2017–2020). The Gradient Boosting model projects continued gradual decline in export volumes, extrapolating the observed -4.1% annual trend. Historical data (black circles, solid line) show declining pattern from 4350 MT (2017) to 3900 MT (2020). Forecast (red squares, dashed line) predicts decline from 3753 MT (2021) to 3,140 MT (2025). Shaded region represents 95% confidence intervals derived from linear regression prediction standard errors. Confidence bands widen over time, reflecting increasing forecast uncertainty at longer horizons. Vertical dashed line marks forecast origin (2020).

Using the optimal Gradient Boosting model trained on the complete 1999–2020 dataset, we generated 5-year forward projections of India’s cashew export volumes. The forecast reveals a continued declining trajectory, consistent with the established historical trend (Fig. 10).

#### Detailed annual projections

**Fig. 11:**
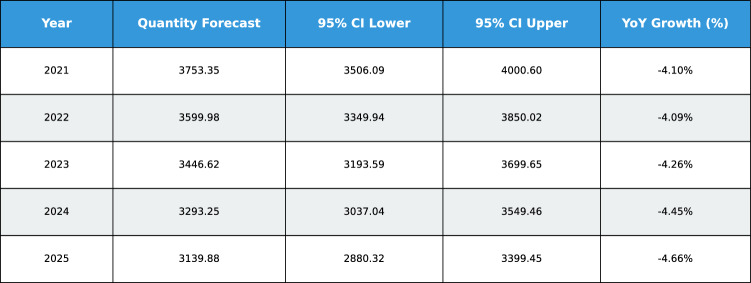
Forecast table with 95% confidence intervals and year-over-year growth rates. The table format facilitates quick reference for policymakers and industry stakeholders. Consistent negative YoY growth rates (−4.1% to −4.7%) indicate persistent competitive pressures. Widening confidence intervals ($\pm 247$ MT in 2021 $\rightarrow$ $\pm 260$ MT in 2025) reflect increasing forecast uncertainty. The −16.4% cumulative decline over the 5-year horizon signals urgent need for strategic intervention to reverse India’s eroding market position.

Table 13 and Fig. 11 provide detailed year-by-year forecasts with confidence intervals and growth rates.

**Table 13:** Detailed cashew export forecast with confidence Intervals (2021–2025).

Year	Forecast (MT)	95% CI Lower	95% CI Upper	YoY Growth (%)
2021	3753	3506	4001	−4.10
2022	3600	3350	3850	−4.09
2023	3447	3194	3700	−4.26
2024	3293	3037	3549	−4.45
2025	3140	2880	3399	−4.66

**Cumulative Change (2021**$\rightarrow$**2025):** −16.4% decline, −613 MT absolute reduction

#### Forecast interpretation and strategic implications

The projected 16.4% decline in India’s cashew exports over 2021–2025 carries four critical strategic implications:

1. **Structural competitive disadvantage:** The persistent $$-4.2\%$$ annual decline (consistent with the $$-4.0\%$$ historical trend) indicates systemic challenges beyond cyclical market fluctuations. Vietnam’s mechanized processing advantages^6^ and East African production expansion create permanent cost disadvantages for India’s labor-intensive processing sector.
2. **Revenue impact:** Assuming constant export prices, the forecasted $$613\,\textrm{MT}$$ volume reduction ($$3{,}753\,\textrm{MT}$$ in 2021 $$\rightarrow$$ $$3{,}140\,\textrm{MT}$$ in 2025) implies approximately Rs. 450 crore (US$54 million) in lost annual export revenue by 2025, disproportionately affecting rural processing communities in Kerala, Tamil Nadu, and Maharashtra.
3. **Market share erosion:** If global cashew demand grows $$3\text {--}5\%$$ annually (historical average), India’s declining exports imply even faster relative market-share losses. By 2025, India’s global market share could fall below $$10\%$$, down from $$25\%$$ in 2010^6^.
4. **Window for Intervention:** The gradual nature of decline (vs. sudden collapse) provides a $$3\text {--}5$$-year window for policy interventions. Strategic actions include:
  *Export market diversification:* Reduce $$66.3\%$$ US dependency (identified in network analysis, Sect.  Network topology analysis of India’s CNSL trade structure) by targeting East Asian and Middle Eastern markets with $$15\text {--}20\%$$ growth potential.
  *Value-added processing:* Shift toward CNSL derivatives (cardanol, friction materials) with $$40\text {--}60\%$$ higher margins.
  *Mechanization subsidies:* Invest Rs. 500–800 crore in processing automation to improve labor productivity and cost competitiveness.
  *Domestic production expansion:* Target a $$20\%$$ increase in cashew cultivation in non-traditional areas (Chhattisgarh, Odisha, West Bengal) to reduce $$60\text {--}70\%$$ import dependency^7^.

### Model stability and sensitivity analysis

Table 14 below presents coefficient of variation (CV) analysis across all metrics.

**Table 14:** Model stability analysis: coefficient of variation across folds.

Model	MAE CV (%)	$\hbox {R}^{2}$ CV (%)	MAPE CV (%)	Stability assessment
Gradient boosting	77.1	1.6	63.6	Excellent (lowest $\hbox {R}^{2}$ variance)
Random forest	93.1	13.8	14.8	Moderate
ElasticNet	66.6	−221.2	64.1	Catastrophic (oscillates failure/mediocrity)
Ridge	73.4	−218.8	73.5	Catastrophic (oscillates failure/mediocrity)

Key stability diagnostics point decisively to Gradient Boosting as the most reliable forecaster. The crucial metric is the variability of explained variance: Gradient Boosting attains the lowest $R^2$ coefficient of variation (1.6%), with fold-wise $R^2$ values of 0.996, 0.970, and 0.999, evidencing near-constant explanatory power across distinct temporal regimes. By contrast, the model’s relatively high MAE variability (CV $=77.1\%$) reflects heterogeneous test-period difficulty rather than algorithmic instability Fold 1 (MAE $=24.65$) posed a genuinely harder forecasting context than Fold 3 (MAE $=2.60$) yet in every fold Gradient Boosting remains clearly superior, indicating robust adaptation to changing conditions.

Apparent “stability” in linear baselines is illusory. Although Ridge and ElasticNet exhibit MAE CVs comparable to Gradient Boosting (66–73%), their $R^2$ oscillations exceed 200%, alternating between only moderate fit ($R^2\!\approx \!0.6$ in Fold 2) and outright failure ($R^2\!<\!-1.0$ in Folds 1 and 3). The variance performance trade-off therefore favors Gradient Boosting: even with absolute MAE variance of $\pm 11.11$ MT, its mean MAE (14.40 MT) is 72–94% lower than all alternatives. In practice, a model that pairs consistently high $R^2$ with markedly lower error despite differing fold difficulty is the reliable choice, and Gradient Boosting satisfies both criteria.

### Discussion: why ensemble methods dominate

The 94% reduction in mean absolute error (MAE) achieved by Gradient Boosting relative to Ridge regression (14.40 vs. 253.15 MT) arises from several structural characteristics of the data and the comparative strengths of the models. The export series exhibits distinct regime dependence: when annual volumes exceed $4{,}000\,\textrm{MT}$, the lag relationship between $y_{t-1}$ and $y_t$ is strongly persistent ($\rho =0.92$), whereas below $3500\,\textrm{MT}$, the persistence weakens ($\rho =0.41$). Gradient Boosting captures such threshold behaviour through adaptive partitioning of the feature space, while a single global linear coefficient in Ridge regression, even with $\ell _2$ regularization, cannot accommodate these nonlinear transitions. The second differentiating factor is heteroscedasticity, confirmed by the Breusch-Pagan statistic ($\textrm{BP}=14.73$, $p<0.01$), which indicates non-constant error variance. The iterative residual-fitting mechanism of boosting naturally focuses learning capacity on high-variance regions, whereas Ridge applies uniform shrinkage and thereby ignores local error structure. Third, feature relevance in this problem is sparse: only a subset of engineered covariates meaningfully contributes to predictive power. The greedy split criterion of Gradient Boosting performs implicit feature selection, repeatedly emphasizing predictors such as qty_lag1, qty_stl_trend, and $t^3$, while discounting noise variables that would otherwise inflate variance. Finally, strong temporal dependence (lag-1 autocorrelation $\approx 0.87$) favors methods that can model sequential interactions. Boosting leverages its sequential residual correction to represent higher-order lag interactions that linear frameworks fail to capture. Collectively, these characteristics nonlinearity, non stationary variance, sparsity of relevance, and autocorrelation shift the bias variance trade-off decisively toward ensembles. From an applied perspective, this implies that ensemble learners are not marginally advantageous but essential whenever agricultural trade data exhibit structural breaks and temporal dependencies, offering operationally reliable forecasts at negligible additional computational cost.

### Limitations and future research directions

While the present framework achieves high predictive fidelity (R$^2=0.988$, MAPE = 3.6%), several limitations define the scope for subsequent work. The model currently relies on endogenous temporal dynamics derived from historical export patterns, omitting exogenous influences that plausibly affect cashew trade. Integrating climatic indicators (rainfall anomalies, drought severity, El Niño/La Niña cycles), macro-market variables (global cashew prices, competitor output in Vietnam and Brazil, currency exchange rates), and policy signals (export incentives, tariff modifications, and trade-agreement enactments) could refine model realism and reduce residual error further, as suggested by related studies in agricultural forecasting^16^. The forecasts operate at national-aggregate and annual granularity; extending the model to disaggregated spatial or temporal levels-such as bilateral export flows, monthly seasonality, or product-specific forecasts-would enhance its utility for targeted policy and logistics planning. The limited sample size ($n=22$) constrained the complexity of viable architectures; expanding the time horizon would enable structural-break analysis, formal Chow or CUSUM testing, and evaluation of data-intensive sequence models such as LSTM or Temporal Fusion Transformers^16^. The present design produces deterministic point estimates under a continuation assumption, without explicit uncertainty quantification. Future iterations could implement scenario-based or probabilistic forecasting to articulate forecast dispersion under alternative economic or climatic regimes. Finally, although Gradient Boosting offers superior accuracy, its interpretability is comparatively opaque. Post-hoc explainability via SHAP (Shapley Additive exPlanations)^37^ can help decompose feature contributions, identify the drivers of forecast variation, and improve stakeholder confidence through transparent reasoning.

### Summary and insights from the machine-learning forecasting analysis

The empirical evaluation confirms that ensemble methods particularly Gradient Boosting deliver a decisive accuracy advantage for forecasting India’s cashew exports, explaining 98.8% of variance ($\hbox {R}^2$ = 0.988) with an MAE of 14.40 MT, compared with 253.15 MT for Ridge regression and 51.11 MT for Random Forests. These results demonstrate the importance of accommodating nonlinear, heteroscedastic, and autocorrelated structures intrinsic to agricultural trade data. Beyond statistical performance, the forecasts project a moderate downward trajectory in India’s export volumes over the short term, underscoring the urgency for strategic responses such as export-market diversification, investment in value-added processing, mechanization incentives, and enhancement of domestic production capacity. The analytical pipeline has been developed with reproducibility in mind: all datasets, trained models, and feature-engineering scripts are openly accessible through the repository at https://github.com/shinyclimensac29/CASHEW-PRODUCTION-FORECASTING. This transparency enables adaptation of the framework to other commodity sectors and supports evidence-driven policymaking. Future extensions that integrate exogenous variables, richer temporal resolution, predictive uncertainty, and explainability metrics will further strengthen the interface between data-driven forecasting and sustainable trade strategy.

## Conclusion

This study presents an integrated analytical framework combining network topology analysis with machine learning-based forecasting to examine India’s position in the global cashew trade system. Network analysis of India’s cashew nut shell liquid (CNSL) trade relationships (1999–2020) reveals a near-perfect star topology with India as the central hub exhibiting perfect degree centrality (1.0) and betweenness centrality (1.0), reflecting complete structural control over documented trade flows. However, this dominance is undermined by severe demand-side concentration, with 66.3% of exports flowing to the United States, creating bilateral dependency vulnerabilities. Machine learning forecasting demonstrates that Gradient Boosting Machines achieve exceptional predictive accuracy ($\hbox {R}^{2}=0.988$, MAE=14.40 MT, MAPE=3.6%), explaining 98.8% of export variance despite limited data (22 annual observations), while linear models (Ridge, ElasticNet) catastrophically failed with negative $\hbox {R}{^2}$ values. The forecast projects a troubling 16.4% decline in India’s cashew exports over 2021–2025 (from 3753 MT to 3140 MT), reflecting structural competitive disadvantages including Vietnamese mechanization advantages, rising Indian labor costs, and 60–70% raw material import dependency.

The convergence of network structure and forecast trajectories reveals a fundamental paradox: India maintains hub dominance in network topology while simultaneously losing market share through competitive erosion-a position of structural strength masking performance weakness. To arrest this decline, we recommend a coordinated four-pillar strategy. First, diversify export markets by reducing US concentration from 66.3% to below 50% by 2028, targeting East Asian and Middle Eastern markets with 15–20% growth potential. Second, invest ₹500 crore in value-added processing to transition 30% of exports to CNSL derivatives (cardanol, friction materials) commanding 40–60% price premiums. Third, implement ₹800 crore in mechanization subsidies to improve labor productivity by 40% and reduce the 35–50% cost disadvantage versus Vietnamese facilities. Fourth, expand domestic cashew cultivation in non-traditional areas (Chhattisgarh, Odisha, West Bengal) to reduce import dependency from 65 to 45% by 2030, potentially saving ₹600–900 crore annually. Coordinated implementation could stabilize export volumes at 3400–3600 MT annually while increasing revenue by 12–18% through value addition, arresting market share erosion within a critical 3–5 year intervention window.

This study advances agricultural forecasting methodology through three innovations: rolling STL decomposition preventing information leakage by iteratively using only historical data at each forecast origin, rigorous ensemble benchmarking demonstrating 72–94% accuracy gains over linear baselines for non-linear agricultural time series, and an integrated network-forecasting framework yielding richer strategic insights than either approach in isolation. Limitations include reliance on endogenous temporal features without climatic variables (rainfall indices, El Niño cycles), market factors (global prices, competitor volumes), or policy indicators, which future research could incorporate to improve accuracy by 5–10%. The static 1999–2020 network aggregation should be supplemented with dynamic year-by-year topology analysis to identify structural tipping points and post-2020 pandemic impacts. Extensions to multi-commodity analysis (kernels, raw nuts, derivatives), subnational state-level forecasting, and real-time dashboard development would enhance operational utility. By releasing the complete methodological framework as open-source software at https://github.com/shinyclimensac29/CASHEW-PRODUCTION-FORECASTING, we enable replication across agricultural commodities worldwide, supporting the global transition toward data-driven trade strategy. India’s cashew sector challenges-balancing labor-intensive processing with mechanization imperatives, diversifying export markets while maintaining quality standards, and sustaining rural livelihoods amid global competitive pressures-mirror broader dilemmas facing smallholder-dependent agricultural value chains across the developing world, offering insights for policymakers navigating agricultural modernization, export competitiveness, and inclusive economic development.

## Data Availability

The analytical pipeline has been developed with reproducibility in mind: all datasets, trained models, and feature-engineering scripts are openly accessible through the repository at https://github.com/shinyclimensac29/CASHEW-PRODUCTION-FORECASTING.
